# The Association Between Alignment to the Breast Cancer Optimal Care Pathways and Patient Survival in Victoria, Australia, 2012–2019: A Retrospective Population‐Based Cohort Study

**DOI:** 10.5694/mja2.70162

**Published:** 2026-03-17

**Authors:** Brandon S. Hao, Juan C. Quiroz, Ian N. Olver, Claire M. Vajdic

**Affiliations:** ^1^ Australian Government Department of Health, Disability and Ageing Canberra Australian Capital Territory Australia; ^2^ Centre for Big Data Research in Health University of New South Wales Sydney New South Wales Australia; ^3^ Adelaide University Adelaide South Australia Australia; ^4^ The Kirby Institute University of New South Wales Sydney New South Wales Australia

**Keywords:** breast neoplasms, guidelines as topic, risk factors, survival analysis, treatment outcome

## Abstract

**Objectives:**

To quantify the association between care alignment to the treatment step of the Cancer Council Victoria and Department of Health Victoria *Optimal care pathways for people with breast cancer* (OCP) (second edition) and survival.

**Design:**

Retrospective population‐based cohort study using the Victorian Cancer Registry and linked administrative health datasets.

**Setting, Participants:**

Adult women diagnosed with invasive, unilateral breast cancer from 1 July 2012 to 31 December 2019 in Victoria, Australia.

**Main Outcome Measures:**

Breast cancer‐specific and overall survival for women whose care did or did not align to the treatment step of the OCP expressed as adjusted hazard ratios. Interaction between OCP alignment and cancer stage at diagnosis was also assessed.

**Results:**

Of 29,591 eligible women, 17,152 (58.0%) were fully aligned, 7086 (23.9%) were partially aligned and 5353 (18.1%) were not aligned to the treatment step of the breast cancer OCP. Median follow‐up was 1481 days (interquartile range, 850–2210 days). Adjusting for measured sociodemographic and clinical factors, OCP treatment alignment was associated with 23% (95% confidence interval [CI], 14%–31%) and 34% (95% CI, 29%–40%) lower risk of death from breast cancer and all causes, respectively, compared with non‐alignment. By cancer stage, OCP alignment was significantly associated with 40% (95% CI, 27%–50%) and 30% (95% CI, 14%–42%) lower risk of breast cancer death for Stage II and III cancers, respectively, and 39% (95% CI, 26%–49%), 49% (95% CI, 42%–55%) and 33% (21%–44%) lower risk of all‐cause death for Stage I, II and III cancers, respectively.

**Conclusions:**

Risk of death was lower for women with breast cancer whose treatment aligned to the OCP compared with women whose treatment did not align. Our findings support the promotion and implementation of the breast cancer OCP.

## Plain Language Summary

**The Known:**

The Cancer Council Victoria and Department of Health Victoria *Optimal care pathways for people with breast cancer* (second edition) describes key elements of best practice cancer care including treatment. While nationally endorsed, there is limited evidence on care alignment in real‐world practice and its associations with survival.

**The New:**

Women with breast cancer whose treatments aligned to the optimal care pathways had 23% and 34% lower risk of breast cancer and all‐cause death, respectively, than patients whose treatments did not align.

**The Implications:**

Our findings highlight the importance of timely best practice breast cancer treatments as well as policies that support routine implementation of the optimal care pathways.

## 1 Introduction

Breast cancer is the second‐highest cause of cancer‐related deaths among Australian women after lung cancer [1]. Although the past three decades saw significant improvements in cancer survival worldwide and in Australia [1, 2], substantial variations in survival remain across clinical and sociodemographic indicators [3, 4, 5]. Recent evidence in breast and other cancers shows further variations associated with alignment to cancer care pathways [6] and timeliness of treatments [7].

Cancer care pathways define best practice, end‐to‐end pathways through the health system that aim to reduce variation in cancer care and improve survival [8, 9]. Cancer care pathways are not clinical practice guidelines. They are a broad health systems approach towards equitable cancer care. The selection of specific therapies remains the remit of clinicians, guided by patient preferences and clinical features.

In Australia, the optimal care pathways (OCP) are nationally endorsed guides that describe key elements of best practice cancer care from prevention to survivorship or end‐of‐life [10]. The 2021 OCP for breast cancer (second edition) [11] outlines recommendations for high‐level treatment pathways and timeframes. Compared with the 2015 first edition [12], the 2021 OCP retained the overarching recommendations on treatments and provided additional commentary on breast cancer stages and subtypes. The routine integration of OCPs, underpinned by the *National OCPs Framework* [13], is a key priority in the 2023 *Australian Cancer Plan*—Australia's 10‐year cancer care reform agenda [14].

Internationally, although individual studies have found positive clinical outcomes associated with alignment to cancer care pathways [15, 16, 17], outcomes were often not comparable between studies [9]. In Australia, most OCP studies focus on feasibility and alignment [18, 19, 20, 21, 22]. Only one Victorian population‐based colon cancer study evaluated clinical outcomes associated with OCP alignment. It found that alignment to the prevention and treatment steps was associated with lower cancer stage at diagnosis and greater survival, respectively [6]. To address this gap in the evaluation of OCPs and outcomes, the present study investigated the relationship between alignment to the breast cancer OCP and survival.

## 2 Methods

A retrospective population‐based cohort study was conducted to quantify the association between alignment to the breast cancer OCP treatment step, and breast cancer‐specific and overall survival in Victoria, Australia, between 2012 and 2019 using linked administrative health data. The study cohort identified adult Victorian women (female sex v. male) registered in the Victorian Cancer Registry (VCR) with invasive, unilateral breast cancer (C50, International Classification of Diseases, tenth revision, Australian modification [23]) between 1 July 2012 and 31 December 2019. The Australian Institute of Health and Welfare (AIHW) performed probabilistic data linkage between Victorian datasets, including admitted care in public and private hospitals (Victorian Admitted Episodes Dataset [VAED]), BreastScreen Victoria and radiation therapy usage data (Victorian Radiotherapy Minimum Data Set), and national ambulatory care (Medicare Benefits Schedule) and prescription medicine dispensing data (Pharmaceutical Benefits Scheme [PBS]) with a linkage rate of 99.7%. The lower cohort cut‐off date was selected to circumvent known under‐identification of prescription medicines dispensed before mid‐2012 in the PBS [24]. Patients with Stage I, II, III or unknown stage disease diagnosed with metastatic cancer (C78, C79) in the VAED within 4 months of VCR diagnosis were recoded as Stage IV [6]. Available data included information on cancer diagnoses and clinical features, other patient medical conditions, patient area‐level socioeconomic condition measured by the Index of Relative Socioeconomic Disadvantage [25], patient remoteness measured by the Modified Monash Model [26], mortality to 30 November 2020, breast screening and treatments. Cancer recurrence data were not available as they are not routinely collected. The study is reported in accordance with the Strengthening the Reporting of Observational Studies in Epidemiology [27] guidelines for cohort studies (checklist provided in Supporting Information).

### 2.1 OCP Alignment

The breast cancer OCP [11] did not describe treatment for patients prescriptively. Instead, it provided broad recommended treatment pathways and timeframes aligned to clinical features such as cancer stage and subtype, allowing nuance in the interpretation of OCP‐aligned treatment (Figure S1). For example, the OCP recommended surgery with curative intent for early‐stage (Stages I/II) and locally advanced (Stage III) cancers but acknowledged neoadjuvant systemic therapy should additionally be considered for locally advanced cancer. For metastatic (Stage IV) cancer, treatment was separately outlined and was not discussed in curative terms. Although the OCP recommended that systemic therapy should be considered for all invasive cancers, it acknowledged that the appropriateness of adjuvant systemic therapy for early cancers requires discussion with patients, recognising that it may not be clinically necessary in all cases.

Criteria for alignment to the OCP treatment step were developed in consultation with an expert medical oncologist experienced in breast cancer treatments and guidelines, with consideration of the OCP's intent and of real‐world oncological practice. Seven broad pathways representing OCP‐aligned treatment were identified.

- Diagnosis → mastectomy
- Diagnosis → breast‐conserving surgery → radiation therapy
- Diagnosis → mastectomy → adjuvant systemic therapy
- Diagnosis → breast‐conserving surgery → adjuvant systemic therapy → radiation therapy
- Diagnosis → neoadjuvant systemic therapy → mastectomy
- Diagnosis → neoadjuvant systemic therapy → breast‐conserving surgery → radiation therapy
- Diagnosis → neoadjuvant systemic therapy → breast‐conserving surgery → adjuvant systemic therapy → radiation therapy

OCPs provide broad treatment recommendations at high‐level therapeutic categories such as surgeries and systemic therapies (chemotherapy, human epidermal growth factor receptor 2 [HER2]‐directed therapy, endocrine therapy). As novel therapies, such as olaparib, became available over the study period, they were accepted as appropriate treatments under the OCP and captured in the data for analysis. The selection of specific therapies—guided by clinical evidence and patient characteristics—remains the role of clinical guidelines, which operate in parallel with the OCP. The high‐level OCP approach addresses the lack of specificity in administrative hospital data about systemic therapies administered to patients.

Women with Stage I/II breast cancer were considered fully aligned to the OCP if their care pathway fully adhered to any of the seven treatment pathways within the indicated timeframes. Stage III cancer patients were considered fully aligned if their care pathway adhered to a treatment pathway that involved systemic therapy (Pathways 3–7). Systemic therapy includes endocrine therapy and chemotherapy. Either or both endocrine therapy and chemotherapy constitute appropriate forms of systemic therapy for hormone receptor‐positive cancer patients. Chemotherapy is the only appropriate systemic therapy for hormone receptor‐negative cancer patients. Due to limited granularity of VAED and Medicare data, HER2‐directed therapies, such as trastuzumab, were recorded as chemotherapy. Patients with metastatic breast cancer were considered fully aligned from initiation of systemic therapy across Pathways 3–7. This meant that Stage IV cancer patients did not need to have breast surgery to be considered fully aligned to the OCP, reflecting that systemic treatments are the mainstay and treatment intent is most often not curative, in line with international guidelines referenced in the OCP [11, 28].

### 2.2 Statistical Analysis

Time‐to‐event analysis was used to quantify the association between alignment to the OCP treatment step and survival. Patient follow‐up began on VCR date of diagnosis (t0) and ended at death (event) or 30 November 2020 (censoring), whichever occurred first. For breast cancer‐specific survival, patients who died from non‐breast cancer causes were censored. Kaplan–Meier and cumulative incidence analyses [29] were conducted to examine baseline survival.

Protecting against immortal time bias, OCP alignment was a binary, time‐varying covariate with all patients starting as not aligned at diagnosis unless they initiated treatment on the day of diagnosis [30]. A detailed explanation of OCP coding rules and time‐varying OCP alignment is presented in Box S1.

Multivariable time‐varying Cox regressions [31] were conducted to quantify the association between OCP alignment and survival in adjusted hazard ratios (aHRs) while controlling for time‐invariant confounders and competing exposures such as cancer stage at diagnosis and age [32]. A directed acyclic graph informed variable selection (Figure S2).

Complementing all‐time survival, the association between OCP alignment with 1‐, 3‐ and 5‐year survival was investigated. As baseline risks of mortality and criteria for OCP alignment differed across cancer stages, post hoc models with a stage‐alignment interaction term were fitted to assess for potential interactions. Pooled likelihood ratio tests were conducted to assess model fit [33]. Subtype–alignment interaction was not assessed as, unlike stage, subtype does not determine the appropriateness of whole pathways—which are the focus of OCPs—but rather the appropriateness of (systemic) therapies within those pathways, which is a clinical consideration. Stepwise regressions were conducted to elucidate the association between cancer subtype and survival.

Data were stored and accessed in a secure remote environment. All analyses were performed using R 4.3.1.

### 2.3 Missing Data and Sensitivity Analyses

Missing data were imputed with multiple imputation with chained equations [34]. Fifty imputations of 30 iterations were performed. To aid performance of the imputation model, survival time, breast cancer‐specific and all‐cause death and treatments received up to 6 months post‐diagnosis were included along with study variables [35].

Recognising that clinicians may have diverse opinions on what constitutes optimal care pathways, sensitivity analyses were conducted to examine the effects of different criteria for OCP alignment on its associations with survival. A complete case analysis and an analysis excluding cases with missing cancer stage or subtype from the original dataset were also conducted.

### 2.4 Ethics Statement

The AIHW Ethics Committee approved the project (EO2015/4/219).

## 3 Results

**FIGURE 1:**
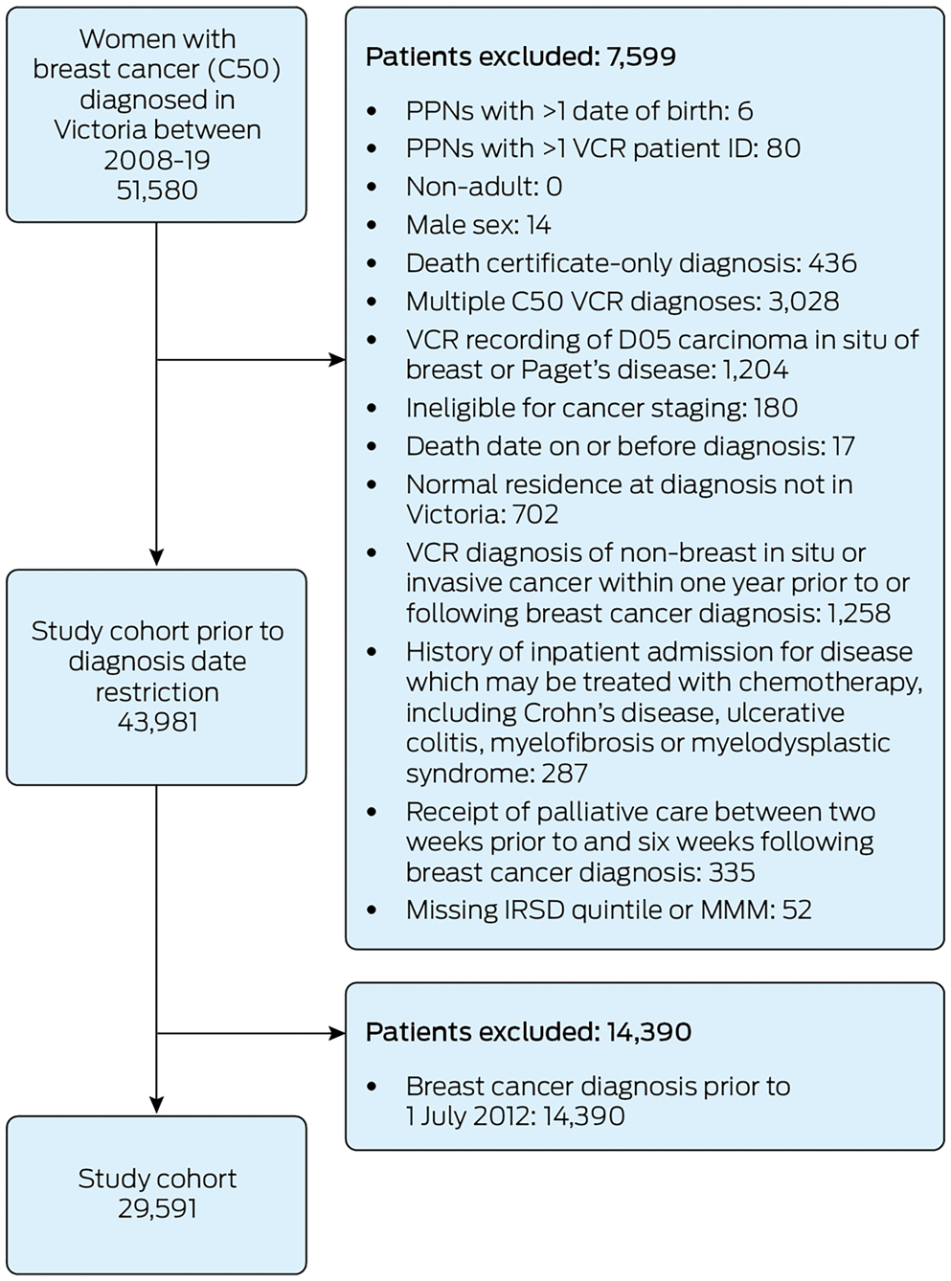
Study cohort selection and patient exclusions. C50, breast cancer [23]; D05, carcinoma in situ of breast [23]; IRSD, Index of Relative Socioeconomic Disadvantage [25]; MMM, Modified Monash Model [26]; PPN, project person number; VCR, Victorian Cancer Registry.

The study cohort included 29,591 women diagnosed with invasive, unilateral breast cancer (Figure 1). At diagnosis, 1171 (4.0%) patients resided in a Statistical Area 2 bordering New South Wales or South Australia. Missing clinical data ranged from 1456 (4.9%) for cancer subtype to 2128 (7.2%) for cancer stage and 4281 (14.5%) for the number of positive lymph nodes. A complete case analysis would have excluded 6704 (22.7%) patients.

Median age at diagnosis was 62 years (interquartile range [IQR], 51–71 years). Median follow‐up was 1481 days (IQR, 850–2210 days). Of 3125 (10.6%) deaths, 1745 (55.8%) had breast cancer registered as the underlying cause. Five‐year breast cancer‐specific and overall survival was 93.1% (95% confidence interval [CI], 92.7%–93.4%) and 87.9% (95% CI, 87.4%–88.3%), respectively. Five‐year survival for each time‐invariant covariate is presented in Table 1.

**TABLE 1:** Time‐invariant study covariates: 5‐year survival by patient sociodemographic characteristics and cancer clinical features.

Characteristic	Count (%)	Five‐year survival (95% CI)	
		Breast cancer‐specific survival	Overall survival
All patients	29,591 (100%)	93.1 (92.7–93.4)	87.9 (87.4–88.3)
Age at diagnosis (years)			
< 50	6239 (21.1%)	94.1 (93.4–94.7)	93.1 (92.3–93.8)
50–64	10,816 (36.6%)	94.7 (94.1–95.1)	92.9 (92.3–93.5)
65–74	7330 (24.8%)	94.9 (94.3–95.5)	91.8 (91.1–92.6)
75+	5206 (17.6%)	86.0 (84.9–87.1)	65.3 (63.7–66.9)
BreastScreen‐detected			
No	20,115 (68.0%)	90.5 (90.1–91.0)	83.6 (83.0–84.3)
Yes	9476 (32.0%)	98.6 (98.3–98.8)	96.9 (96.5–97.3)
Culturally and linguistically diverse status^a^			
No	27,642 (93.4%)	93.3 (93.0–93.6)	88.2 (87.8–88.7)
Yes	1949 (6.6%)	89.9 (88.3–91.4)	82.3 (80.3–84.4)
Charlson Comorbidity Index score^b^ [36]			
0	28,022 (94.7%)	93.5 (93.2–93.9)	89.2 (88.8–89.6)
1+	1569 (5.3%)	85.2 (83.1–87.1)	63.3 (60.5–66.2)
IRSD quintile of patient residential area at diagnosis [25]			
5—Least disadvantaged	6700 (22.6%)	95.1 (94.4–95.7)	91.3 (90.5–92.1)
4	5992 (20.2%)	94.7 (94.0–95.3)	90.4 (89.6–91.3)
3	5740 (19.4%)	92.9 (92.1–93.7)	88.2 (87.2–89.2)
2	5570 (18.8%)	91.7 (90.9–92.5)	85.6 (84.5–86.7)
1—Most disadvantaged	5589 (18.9%)	90.5 (89.6–91.4)	82.9 (81.7–84.1)
MMM remoteness of patient residential area at diagnosis [26]			
1—Metropolitan areas	21,735 (73.5%)	93.3 (92.9–93.7)	88.3 (87.8–88.9)
2—Regional centres	2191 (7.4%)	93.5 (92.2–94.6)	87.9 (86.3–89.6)
3—Large rural towns	1650 (5.6%)	92.5 (91.0–93.9)	86.9 (85.0–88.8)
4—Medium rural towns	1618 (5.5%)	91.6 (89.9–93.1)	84.9 (82.9–87.0)
5+—Small rural and remote	2397 (8.1%)	91.8 (90.5–93.0)	85.9 (84.3–87.6)
Year of diagnosis			
2012	1727 (5.8%)	90.9 (89.5–92.2)	85.5 (83.8–87.1)
2013	3703 (12.5%)	92.1 (91.2–93.0)	86.8 (85.7–87.9)
2014	3875 (13.1%)	91.5 (90.6–92.3)	86.3 (85.3–87.4)
2015	3880 (13.1%)	92.8 (91.9–93.6)	87.8 (86.8–88.8)
2016	3956 (13.4%)	—	—
2017	4074 (13.8%)	—	—
2018	4163 (14.1%)	—	—
2019	4213 (14.2%)	—	—
AJCC summary stage at diagnosis			
I	12,679 (42.8%)	99.1 (98.9–99.3)	96.5 (96.1–96.8)
II	10,768 (36.4%)	95.2 (94.7–95.7)	90.4 (89.7–91.1)
III	2717 (9.2%)	84.6 (83.0–86.1)	78.9 (77.1–80.7)
IV	1299 (4.4%)	53.0 (49.9–56.2)	44.0 (40.9–47.4)
Unknown	2128 (7.2%)	78.3 (75.8–80.7)	54.9 (51.9–58.1)
Tumour grade			
I—Well differentiated	4437 (15.0%)	99.4 (99.1–99.6)	95.6 (94.9–96.3)
II—Moderately differentiated	12,395 (41.9%)	96.5 (96.1–96.9)	91.7 (91.1–92.3)
III—Poorly differentiated	9507 (32.1%)	89.4 (88.7–90.1)	84.3 (83.5–85.2)
Unknown	3252 (11.0%)	81.7 (80.1–83.2)	72.1 (70.3–74.0)
Breast cancer subtype^c^			
Luminal A	21,933 (74.1%)	95.2 (94.9–95.5)	90.1 (89.6–90.6)
Luminal B (HER2‐positive)	2549 (8.6%)	93.6 (92.5–94.7)	90.4 (89.1–91.8)
HER2‐amplified	1161 (3.9%)	91.5 (89.5–93.3)	85.5 (83.1–87.9)
Triple negative	2492 (8.4%)	82.9 (81.2–84.5)	77.4 (75.6–79.3)
Unknown	1456 (4.9%)	79.7 (77.4–81.9)	70.1 (67.5–72.8)
Tumour size (mm)			
< 15	9559 (32.3%)	98.5 (98.2–98.8)	96.4 (95.9–96.8)
15–34	12,214 (41.3%)	95.9 (95.4–96.3)	91.6 (91.0–92.2)
35+	4170 (14.1%)	88.4 (87.2–89.5)	81.7 (80.3–83.1)
Unknown	3648 (12.3%)	73.2 (71.5–75.0)	57.5 (55.5–59.5)
Histology [37]			
Ductal	23,833 (80.5%)	93.7 (93.3–94.0)	88.8 (88.4–89.3)
Lobular	3539 (12.0%)	92.8 (91.7–93.7)	87.0 (85.6–88.3)
Other	2219 (7.5%)	87.2 (85.6–88.7)	78.8 (76.9–80.8)
Number of positive lymph nodes			
0	17,240 (58.3%)	98.2 (97.9–98.4)	95.2 (94.8–95.6)
1–3	5900 (19.9%)	95.1 (94.5–95.8)	91.4 (90.5–92.3)
4+	2170 (7.3%)	83.0 (81.1–84.8)	77.7 (75.7–79.7)
Unknown	4281 (14.5%)	73.3 (71.7–75.0)	55.5 (53.7–57.5)

*Note:* Five‐year breast cancer‐specific and overall survival were calculated using the complement of cumulative incidence function and the Kaplan–Meier estimator, respectively.

Abbreviations: AJCC, American Joint Committee on Cancer; CI, confidence interval; HER2, human epidermal growth factor receptor 2; IRSD, Index of Relative Socioeconomic Disadvantage; MMM, Modified Monash Model.

^a^ Defined as interpreter required during hospital care episode or patient preferred language is language other than English.

^b^ Extracted using non‐cancer diagnoses from inpatient hospital admissions between 1‐year prior and 30 days post‐breast cancer diagnosis in the Victorian Cancer Registry.

^c^ Oestrogen, progesterone and human epidermal growth factor receptor 2 status are available in the Victoria Cancer Registry as positive, negative or unknown. Breast cancer was: (1) luminal A if either oestrogen or progesterone receptor‐positive (hormone receptor‐positive) and human epidermal growth factor receptor 2‐negative; (2) luminal B if hormone receptor‐positive and human epidermal growth factor receptor 2‐positive; (3) HER2‐amplified if hormone receptor‐negative and human epidermal growth factor receptor 2‐positive; (4) triple negative if hormone receptor‐negative and human epidermal growth factor receptor 2‐negative; and (5) unknown if cancer subtype could not be determined from above. Analyses were conducted on the original data.

### 3.1 OCP Alignment and Patient Characteristics

Taking the mean across imputations, 17,152 (58.0%) patients were fully aligned, 7086 (23.9%) patients partially aligned, and 5353 (18.1%) patients never aligned to the OCP treatment step at the end of follow‐up. Of the patients in border areas, 353 of 1171 (30.1%) never aligned to the OCP compared with 5000 of 28,420 (17.6%) non‐border patients.

Patients with the least area‐level socioeconomic disadvantage and patients with triple negative cancer had the highest and lowest rate of full alignment (4367 of 6700 [65.2%] vs. 1165 of 2665 [43.7%]; Table 2), respectively. The rate of full alignment decreased almost linearly across area‐level socioeconomic disadvantage quintiles (least disadvantaged, 4367 of 6700 [65.2%] to most disadvantaged, 2801 of 5589 [50.1%]; Table 2).

**TABLE 2:** Mean optimal care pathway alignment and association with survival by study covariates.

Characteristic	OCP Alignment			aHR (95% CI)	
	Full	Partial	Never	Breast cancer‐specific death	All‐cause (overall) death
All patients	17,152 (58.0%)	7086 (23.9%)	5353 (18.1%)		
Age at diagnosis (years)					
< 50	3703 (59.3%)	1514 (24.3%)	1022 (16.4%)	0.77 (0.67–0.90)	0.71 (0.62–0.80)
50–64	6528 (60.4%)	2278 (21.1%)	2010 (18.6%)	1.00 (reference)	1.00 (reference)
65–74	4426 (60.4%)	1606 (21.9%)	1298 (17.7%)	1.14 (0.98–1.32)	1.33 (1.18–1.50)
75+	2496 (48.0%)	1688 (32.4%)	1021 (19.6%)	1.87 (1.63–2.15)	3.60 (3.24–3.99)
BreastScreen‐detected					
No	11,446 (56.9%)	5106 (25.4%)	3563 (17.7%)	1.00 (reference)	1.00 (reference)
Yes	5706 (60.2%)	1980 (20.9%)	1790 (18.9%)	0.38 (0.31–0.47)	0.42 (0.36–0.48)
Culturally and linguistically diverse status					
No	16,166 (58.5%)	6591 (23.8%)	4885 (17.7%)	1.00 (reference)	1.00 (reference)
Yes	986 (50.6%)	495 (25.4%)	468 (24.0%)	0.97 (0.80–1.17)	0.86 (0.74–1.00)
Charlson Comorbidity Index score [35]					
0	16,403 (58.5%)	6628 (23.7%)	4991 (17.8%)	1.00 (reference)	1.00 (reference)
1+	750 (47.8%)	457 (29.2%)	362 (23.1%)	1.41 (1.17–1.70)	1.94 (1.71–2.21)
IRSD quintile of patient residential area at diagnosis					
5 (least disadvantaged)	4367 (65.2%)	1557 (23.2%)	776 (11.6%)	1.00 (reference)	1.00 (reference)
4	3647 (60.9%)	1344 (22.4%)	1001 (16.7%)	0.94 (0.79–1.13)	0.99 (0.87–1.13)
3	3309 (57.6%)	1386 (24.2%)	1045 (18.2%)	1.21 (1.02–1.44)	1.18 (1.04–1.34)
2	3028 (54.4%)	1384 (24.8%)	1158 (20.8%)	1.23 (1.04–1.46)	1.25 (1.10–1.41)
1 (most disadvantaged)	2801 (50.1%)	1415 (25.3%)	1373 (24.6%)	1.23 (1.04–1.46)	1.25 (1.10–1.42)
MMM remoteness of patient residential area at diagnosis					
1	12,945 (59.6%)	5224 (24.0%)	3566 (16.4%)	1.00 (reference)	1.00 (reference)
2	1231 (56.2%)	480 (21.9%)	480 (21.9%)	0.93 (0.76–1.14)	0.97 (0.82–1.14)
3	873 (52.9%)	430 (26.1%)	347 (21.0%)	1.16 (0.92–1.46)	1.11 (0.93–1.33)
4	836 (51.7%)	389 (24.1%)	393 (24.3%)	1.07 (0.85–1.34)	0.97 (0.81–1.15)
5+	1268 (52.9%)	563 (23.5%)	566 (23.6%)	1.14 (0.96–1.37)	1.09 (0.95–1.26)
Year of diagnosis					
2012	987 (57.1%)	463 (26.8%)	277 (16.1%)	1.43 (1.16–1.78)	1.37 (1.16–1.62)
2013	2224 (60.1%)	898 (24.2%)	581 (15.7%)	1.22 (1.02–1.47)	1.17 (1.02–1.35)
2014	2366 (61.1%)	902 (23.3%)	607 (15.6%)	1.32 (1.10–1.59)	1.23 (1.07–1.43)
2015	2323 (59.9%)	949 (24.5%)	608 (15.7%)	1.04 (0.86–1.27)	1.05 (0.90–1.22)
2016	2276 (57.5%)	1005 (25.4%)	675 (17.1%)	1.00 (reference)	1.00 (reference)
2017	2331 (57.2%)	938 (23.0%)	805 (19.7%)	0.91 (0.73–1.12)	0.95 (0.80–1.11)
2018	2298 (55.2%)	1000 (24.0%)	865 (20.8%)	0.73 (0.56–0.93)	0.85 (0.70–1.03)
2019	2349 (55.7%)	930 (22.1%)	934 (22.2%)	0.41 (0.28–0.60)	0.81 (0.64–1.02)
AJCC summary stage at diagnosis					
I	8048 (61.1%)	3003 (22.8%)	2115 (16.1%)	1.00 (reference)	1.00 (reference)
II	6711 (57.3%)	2887 (24.6%)	2117 (18.1%)	2.80 (2.16–3.64)	1.69 (1.45–1.97)
III	1396 (44.7%)	1091 (35.0%)	632 (20.3%)	6.08 (4.30–8.60)	2.60 (2.05–3.31)
IV	998 (62.7%)	104 (6.6%)	489 (30.7%)	26.78 (19.94–35.96)	9.61 (7.96–11.61)
Tumour grade					
I	2875 (59.6%)	1095 (22.7%)	855 (17.7%)	1.00 (reference)	1.00 (reference)
II	8308 (60.4%)	2978 (21.6%)	2469 (18.0%)	1.92 (1.33–2.78)	1.05 (0.89–1.24)
III	5969 (54.2%)	3013 (27.4%)	2028 (18.4%)	3.65 (2.51–5.29)	1.47 (1.23–1.75)
Breast cancer subtype					
Luminal A	14,101 (61.2%)	4942 (21.5%)	3984 (17.3%)	1.00 (reference)	1.00 (reference)
Luminal B (HER2‐positive)	1261 (47.0%)	902 (33.6%)	521 (19.4%)	0.76 (0.63–0.92)	0.78 (0.67–0.91)
HER2‐amplified	625 (51.5%)	321 (26.5%)	267 (22.0%)	0.96 (0.75–1.23)	1.08 (0.88–1.32)
Triple negative	1165 (43.7%)	919 (34.5%)	581 (21.8%)	2.56 (2.18–2.99)	2.01 (1.76–2.30)
Tumour size (mm)					
< 15	6176 (58.7%)	2487 (23.6%)	1858 (17.7%)	1.00 (reference)	1.00 (reference)
15–34	8253 (58.0%)	3456 (24.3%)	2510 (17.7%)	1.21 (0.98–1.50)	1.19 (1.03–1.39)
35+	2724 (56.1%)	1144 (23.6%)	984 (20.3%)	1.40 (1.12–1.76)	1.39 (1.17–1.64)
Histology					
Ductal	13,744 (57.7%)	5868 (24.6%)	4220 (17.7%)	1.00 (reference)	1.00 (reference)
Lobular	2244 (63.4%)	681 (19.3%)	614 (17.4%)	1.10 (0.93–1.30)	1.05 (0.93–1.18)
Other	1165 (52.5%)	536 (24.2%)	518 (23.4%)	1.42 (1.19–1.69)	1.31 (1.14–1.51)
Number of positive lymph nodes					
0	11,545 (60.4%)	4344 (22.7%)	3236 (16.9%)	1.00 (reference)	1.00 (reference)
1–3	4258 (56.0%)	1843 (24.2%)	1502 (19.8%)	1.52 (1.27–1.81)	1.32 (1.16–1.49)
4+	1349 (47.1%)	899 (31.4%)	615 (21.5%)	1.89 (1.46–2.44)	1.63 (1.33–2.01)

*Note:* The outcome was death. Hazard ratios were taken from the fully adjusted all‐time non‐interaction model. Analyses were conducted and pooled across 50 imputations. Mean counts across imputations were rounded to the nearest whole number. Due to rounding error, cells may not add up to the total. Proportions presented are within‐group (row‐wise).

Abbreviations: aHR, adjusted hazard ratios; AJCC, American Joint Committee on Cancer; CI, confidence interval; HER2, human epidermal growth factor receptor 2; IRSD, Index of Relative Socioeconomic Disadvantage; MMM, Modified Monash Model; OCP, optimal care pathway.

### 3.2 Associations With Survival

**FIGURE 2:**
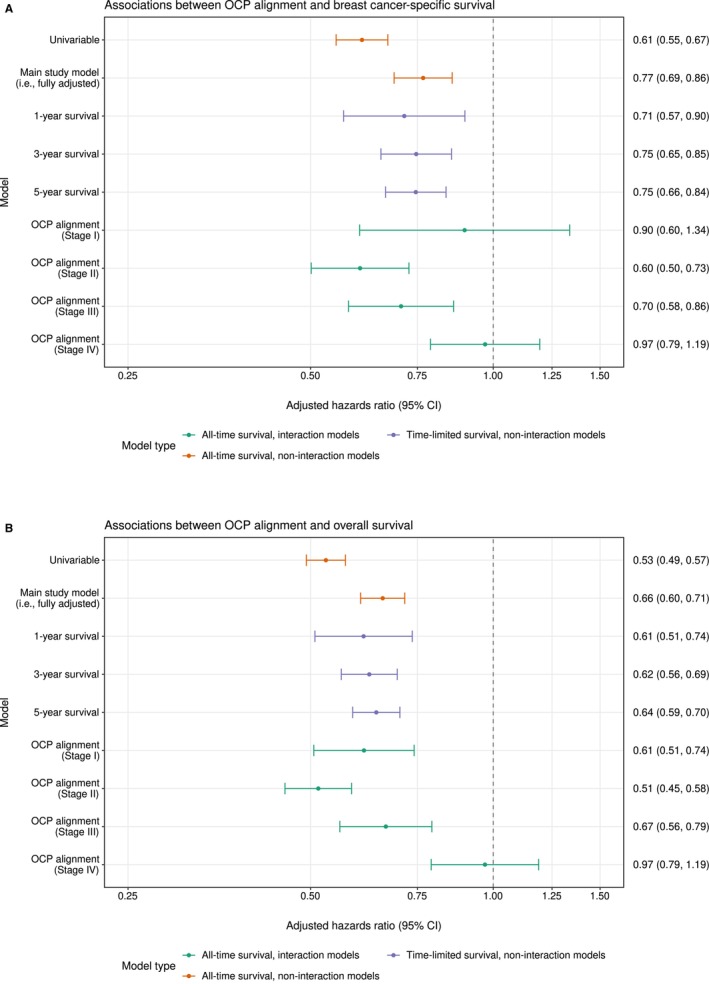
Associations between optimal care pathway alignment and breast cancer‐specific survival (A) and overall survival (B) across model specifications. CI, confidence interval; OCP, optimal care pathway. (A, B) Adjusted hazard ratios, the associations between optimal care pathway alignment and breast cancer‐specific and overall survival across model specifications, respectively. The outcome was death. All models were fully adjusted with time‐invariant study variables from Table 2, except the univariable model. Confidence intervals for the joint marginal effects of optimal care pathway alignment across cancer stages were manually calculated from the covariance matrix [38]. Analyses were conducted and pooled across 50 imputations.

After adjustment, alignment to the OCP treatment step was associated with 23% (95% CI, 14%–31%) lower risk of breast cancer death and 34% (95% CI, 29%–40%) lower risk of all‐cause death compared with non‐alignment (Figure 2). Improved outcomes associated with OCP alignment appeared relatively stable across survival durations with broad overlap between confidence intervals (Figure 2). aHRs and cumulative incidence curves for time‐invariant covariates are presented in Table 2 and Figure S3. Stepwise regression revealed that at baseline, non‐luminal A subtypes were associated with worse breast cancer‐specific survival compared to luminal A cancer (Table S1).

The association between OCP alignment and survival varied by stage at diagnosis (Figure 2), and the addition of stage‐alignment interaction resulted in better model fit for both breast cancer‐specific death (*p* = 0.003) and all‐cause death (*p* < 0.001). OCP alignment was associated with 40% (95% CI, 27%–50%) and 30% (95% CI, 14%–42%) lower risks of breast cancer death for Stage II and III cancers, respectively. OCP alignment was not associated with breast cancer‐specific survival for Stage I and IV cancers. OCP alignment was associated with 39% (95% CI, 26%–49%), 49% (95% CI, 42%–55%) and 33% (95% CI, 21%–44%) lower risks of all‐cause death for Stage I, II and III cancers, respectively, and there was no association for Stage IV cancers.

All sensitivity analyses on imputed data yielded a significant association between OCP alignment and improved survival for the all‐time non‐interaction model (Table S2). Under the OCP criteria that patients with Stage II/III/IV cancers should adhere to a treatment pathway involving systemic therapy (sensitivity analysis 2), OCP alignment was not associated with breast cancer‐specific survival for Stage II cancers (aHR, 0.83; 95% CI, 0.68–1.01). OCP alignment was not associated with breast cancer‐specific survival when cases with missing cancer stage or subtype were excluded (aHR, 0.90; 95% CI, 0.80–1.02).

## 4 Discussion

Examining the records of nearly 30,000 Victorian women with breast cancer diagnosed between 2012 and 2019, we found that alignment to the high‐level OCP treatment type and timing was associated with significantly lower risks of breast cancer‐specific death and all‐cause death compared with non‐alignment. These associations were relatively stable over time since diagnosis, but varied by cancer stage at diagnosis. Overall, our population‐level findings in Victoria point to a survival benefit associated with alignment to the OCP after accounting for measured potential confounding factors. The findings reaffirm the importance of timely treatment [7] and provide an impetus to promote treatment aligned to the OCP.

In post hoc analyses, there was no association between OCP alignment and breast cancer‐specific survival for Stage I cancers, potentially due to very few breast cancer deaths within this cohort (observed 5‐year breast cancer‐specific survival exceeded 99%). OCP alignment was associated with 40% and 30% lower risk of breast cancer death for Stage II and III cancers, respectively, and these results are likely clinically significant. For overall survival, the association with OCP alignment is also likely clinically significant among Stage I, II and III cancers. For Stage IV cancers, OCP alignment does not appear to be associated with improved survival, potentially owing to high baseline risk of mortality among this cohort.

The point estimates for OCP alignment were stronger for overall survival than breast cancer‐specific survival for all women and for women with Stage I or II cancer whose risk of breast cancer death is lower. However, the confidence intervals overlap, suggesting the differences may not be significant. Nevertheless, a potentially greater association between OCP alignment and overall survival than breast cancer‐specific survival may indicate residual confounding or an association with healthcare pathways beyond cancer. As OCP alignment accounts for both receipt and timeliness of treatments, it may be that patients who receive OCP‐aligned care also receive more optimal care in non‐cancer domains. Although the study attempted to adjust for confounding using area‐level indices of socioeconomic status and health resource distribution, residual confounding and confounding from unmeasured patient‐, community‐, health service‐ and health system‐level factors are likely. Indeed, breast cancer treatment intervals have been associated with a range of factors, some of which were not measured in the present study and may be associated with greater health‐seeking behaviours for non‐communicable diseases more generally, such as patient private health insurance status and person‐level income [7, 39].

Despite further investigation needed on what OCP alignment may be measuring, it is clear that OCP alignment is associated with better survival, particularly for Stage III patients whose risk of death from breast cancer is higher. For these patients, the associations of OCP alignment with breast cancer‐specific and overall survival converge, indicating minimal to no additional survival benefit for non‐breast cancer causes. From our adjusted estimates, breast cancer clinical features were consistently more strongly associated with breast cancer‐specific survival, whereas older age and the presence of other non‐cancer medical conditions were more strongly associated with overall survival. These patterns align with expectations, lending validation to the robustness of our data, including the imputation approach.

### 4.1 Implications for Australian Cancer Care Policy

The policy implications of our findings are manifold. Firstly, our study complements the 2019 Victorian study [6] that found an overall survival benefit associated with alignment to the diagnosis and treatment steps of the colorectal cancer OCP (aHR_non‐aligned vs. aligned_, 1.23; 95% CI, 1.13–1.35). Additionally, we provide a new approach for ascribing alignment to the treatment step of an OCP. In contrast to the Victorian colon cancer study, which assessed alignment in discrete segments [6], we assessed alignment in one continuum. Together, in line with the *National OCPs Framework* and the *Australian Cancer Plan* [12, 13], our study strengthens the evidence base for the routine implementation of OCPs and provides a method for monitoring and evaluating the effect of their implementation on patient outcomes using only registry and administrative data.

Having a reproducible and quantifiable indicator of cancer care coordination is critical for cancer policy development. To this end, measurement of OCP alignment could potentially be used as a gauge of health system performance and cancer care equity at local, regional and national levels. Such an indicator could help identify population groups at risk of experiencing cancer care inequities, as in the present study, and uncover potential barriers to care access. Ultimately, the indicators could inform the development of targeted policies, practices and incentives for delivering the OCPs and improving patient outcomes.

Lastly, our results on overall survival, in addition to breast cancer‐specific survival, despite justifying further investigation, offer a reminder of the importance of holistic care. Both the Australian Cancer Plan and OCPs explicitly recognise the value of integrated multidisciplinary care across the cancer continuum beyond cancer‐specific care, which may involve general health promotion and effective management of non‐cancer conditions. Indeed, a comprehensive cancer care system should incorporate a range of approaches for promoting patient outcomes—including encouraging alignment to the OCP and other relevant healthcare pathways.

### 4.2 Limitations

Multiple imputation was used and missingness at random was assumed. However, in our study, patients with missing clinical information often experienced the worst survival, indicating potential missingness not at random at baseline. Although survival and treatment information were included in the imputation to aid performance and satisfy the assumption of missingness at random, bias may remain.

As the median follow‐up (1481 days) was relatively short for a study on breast cancer and survival, our estimates may be less representative for longer‐term survival, although our series of time‐restricted and all‐time analyses show that the associations between OCP alignment and survival were relatively stable over time. Our study could not censor those who emigrated from Victoria. The under‐ascertainment of in‐hospital treatments across borders is likely not trivial [40]. However, since survival is high in our cohort, under‐assignment of OCP‐aligned time for emigrated and border patients is likely to lead to under‐estimations of the associations between OCP alignment and survival.

Although not necessarily a limitation, we could not assess the reasons that care was unaligned. Non‐alignment can include health system and non‐health system reasons. Inequitable access to care and therapeutic toxicities can cause unplanned interruptions to treatment protocols, which might otherwise have aligned with the OCP [41]. Nevertheless, under its core principles of safe and quality care (Principal 2) and multidisciplinary care (Principal 3), the OCP stipulates safe and effective care and ‘access to the full therapeutic range for all patients, regardless of geographical remoteness or size of institution’. From this perspective, non‐alignment to the OCP for whatever reason, reveals unmet needs, potential barriers to equity and opportunities to bolster patient outcomes.

## 5 Conclusion

About six in ten women diagnosed with breast cancer in Victoria between 2012 and 2019 received fully OCP‐aligned treatment and alignment was associated with lower risks of breast cancer‐specific death and all‐cause death. These associations were relatively stable over time since diagnosis and varied across cancer stages. The OCP describes a standard of care that should be available to all patients. Our findings encourage the promotion and uptake of the OCP.

## Author Contributions

**Brandon S. Hao:** conceptualisation, methodology, formal analysis, writing – original draft, writing – review and editing. **Juan C. Quiroz:** conceptualisation, methodology, supervision, writing – review and editing. **Ian N. Olver:** methodology, writing – review and editing. **Claire M. Vajdic:** conceptualisation, methodology, supervision, writing – review and editing.

## Funding

This study was funded by the Australian Government Department of Health, Disability and Ageing as part of its business‐as‐usual research activities. The Australian Government Department of Health, Disability and Ageing funded the data linkage for the study and provided support in relation to project administration, methodology development and manuscript feedback.

## Disclosure

Not commissioned; externally peer reviewed.

## Conflicts of Interest

Brandon Hao holds ongoing employment as a data scientist at the Australian Government Department of Health, Disability and Ageing, which also funded this study as part of its business‐as‐usual research activities. Brandon Hao receives a salary in his role at the Department of Health, Disability and Ageing.

## Supporting information


**Data S1:** mja270162‐sup‐0001‐supinfo.pdf.

**Data S2:** mja270162‐sup‐0002‐supinfo.pdf.

## Data Availability

In keeping with Australian privacy and confidentiality legislation, the individual person‐level data accessed and used for this project are not publicly available and cannot be shared by the authors under the conditions of ethical approval for the research.
