# Supplementary material for: The Association Between Alignment to the Breast Cancer Optimal Care Pathways and Patient Survival in Victoria, Australia, 2012–2019: A Retrospective Population‐Based Cohort Study
Source: Med J Aust. 2026 Mar 17;224(3):e70162. doi: 10.5694/mja2.70162 (PMC12996730; doi:10.5694/mja2.70162)
Supplement: Supplementary file 1 — Data S1: mja270162‐sup‐0001‐supinfo.pdf. [file MJA2-224-0-s001.pdf]

The Medical Journal of Australia

Supporting Information

Supplementary material

This appendix was part of the submitted manuscript and has been peer reviewed.
It is posted as supplied by the authors.

Appendix to: Hao BS, Quiroz JC, Olver IN, Vajdic CM. The association between alignment to the breast cancer Optimal Care Pathways and patient survival in Victoria, Australia, 2012-2019: a retrospective population-based cohort study. *Med J Aust* 2026; doi: 10.5694/mja2.70162.

Figure 1. Treatment pathways and action timeframes for breast cancer optimal care pathways-aligned care.

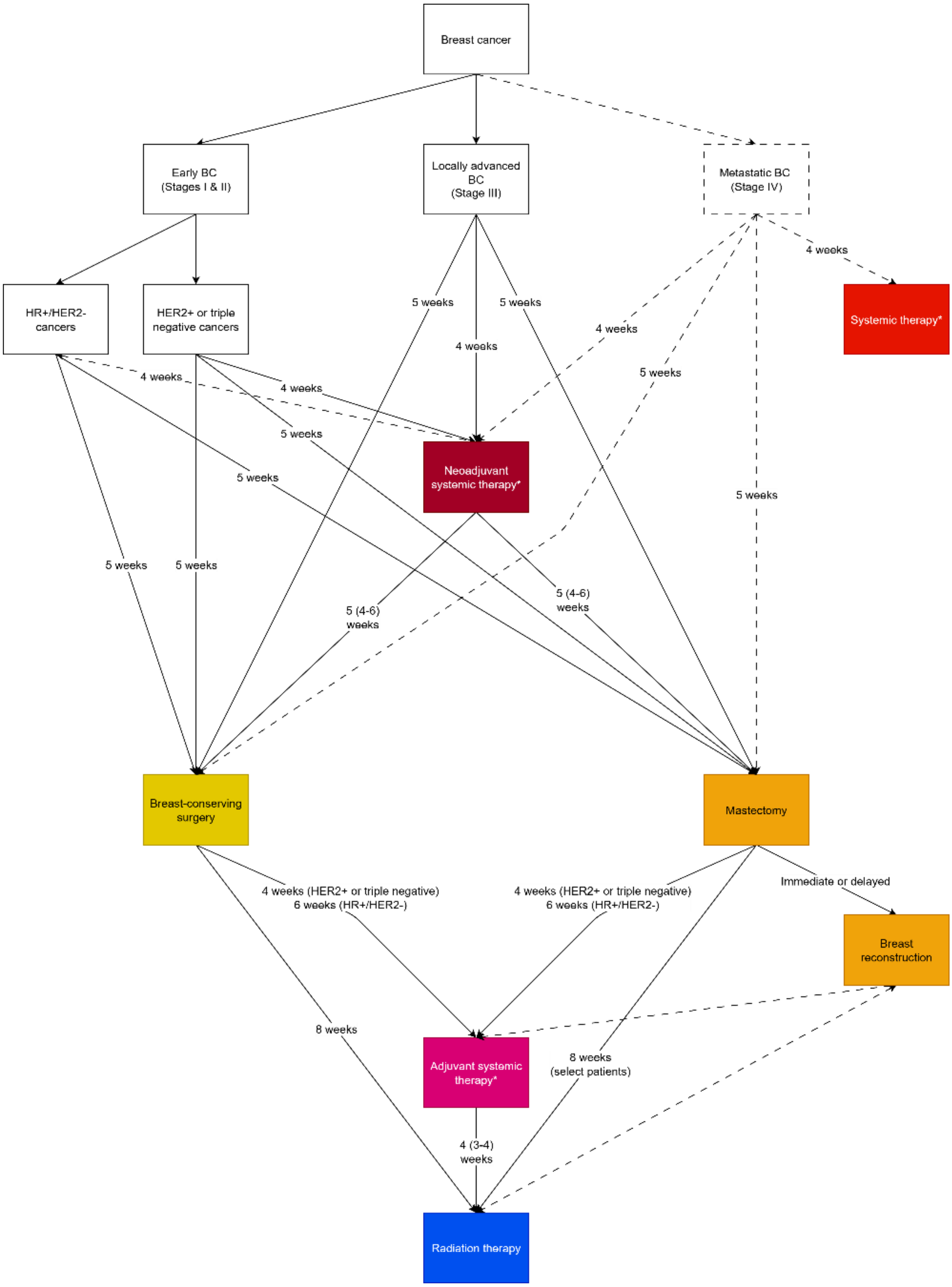

BC = breast cancer; HR+ = hormone receptor-positive; HER2- = human epidermal growth factor receptor 2-negative; HER2+ = human epidermal growth factor receptor 2-positive. * Systemic therapy includes endocrine therapy and chemotherapy, the appropriateness of which depends on patient breast cancer subtype. Solid lines indicate pathway and timeframe vectors explicitly noted under the *Optimal care pathways for people with breast cancer, second edition* (1). Dashed lines indicate pathway and timeframe vectors determined in consultation with an expert medical oncologist and with regards to the intent of the optimal care pathways. For some treatment intervals such as neoadjuvant systemic therapy to surgery, the optimal care pathways outlined a time range instead of a fixed timeframe. For these intervals, the timeframe used in the study to determine cut-off is captured in text and the verbatim time range outlined in the optimal care pathways is captured in parentheses.

Box 1. Detailed OCP coding rules.

All coding procedures were developed in consultation with an expert medical oncologist.

OCP alignment criteria

A patient's treatment was classified as aligned to the OCP if they initiated each treatment of a stage-appropriate pathway within the OCP timeframes. An illustration of patients traversing through the diagnosis → mastectomy → adjuvant systemic therapy treatment pathway is presented below. To protect against immortal time bias, all patients start as "not aligned" at diagnosis unless they initiated treatment on the day of diagnosis.

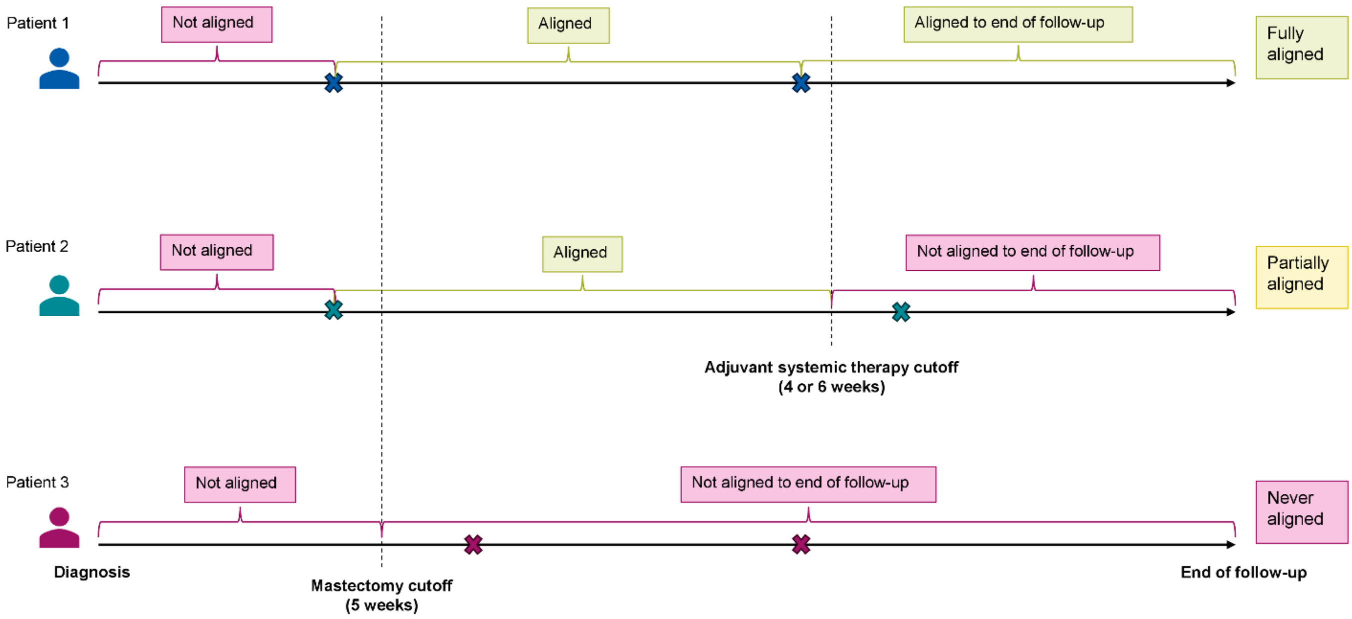

Patient 1 fully aligned to the treatments and timeframes of the mastectomy and adjuvant systemic therapy pathway as they received both treatments prior to their cut-off dates. Thus, patient 1 was considered OCP-aligned from the date they initiated the first treatment of the pathway (mastectomy) to the end of follow-up.

Conversely, patient 2's care initially aligned to the OCP (mastectomy) but subsequently failed to meet timeframes for a later step in the pathway (adjuvant systemic therapy). Thus, patient 2 was considered OCP-aligned for the time between their mastectomy to the cut-off date for the subsequent adjuvant systemic therapy, then not aligned from the cut-off date to the end of follow-up. Patients who changed status from OCP-aligned to not aligned could not become OCP-aligned again.

Lastly, patient 3 did not receive the first treatment of the pathway (mastectomy) within the indicated timeframes and was thus considered not aligned from diagnosis to the end of follow-up.

Attribution of OCP alignment time

A patient's care could be aligned to multiple stage-appropriate pathways. For fully OCP-aligned patients, their OCP alignment time was calculated from the earliest date on which they became OCP-aligned. For partially

OCP-aligned patients, their OCP alignment time was taken from the pathway in which they were OCP-aligned for the longest duration.

Timeframe cut-offs for subsequent treatments in a pathway

For determining cut-off dates for subsequent treatments in a pathway, the OCP-indicated time interval was added to the date of the previous treatment. However, complexities arose where patients received consecutive occurrences of the same treatment, which could reasonably be grouped together as an extended episode of treatment. For example, a patient could receive multiple surgeries as part of local treatment, including a series of breast-conserving surgeries (excisions) and later mastectomy. Additionally, while neoadjuvant and adjuvant systemic therapies typically last up to six months, the duration of systemic treatments could vary between patients (2, 3). In these scenarios, consecutive treatments were grouped and the date of the last treatment was used to calculate the subsequent treatment cut-off date. This is discussed in more detail in the following section.

Extended episodes of treatment

Multiple breast surgeries (either breast-conserving surgery or mastectomy), which occurred within 28 days of the previous surgery were treated as one extended surgery episode. In this scenario, the date of the last surgery was used to calculate the cut-off date for the subsequent treatment. If the extended surgery episode included mastectomy, then the patient was classified as having received mastectomy regardless of its temporal position within the sequence.

For systemic therapies (both neoadjuvant and adjuvant), patients were followed up for up to six months (180 days) from the date they initiated chemotherapy and/or endocrine therapy. The last date of systemic therapy administration within the six-month time window was used to calculate the cut-off date for the subsequent treatment after systemic therapy (4). Where hormone receptor-positive breast cancer patients received both endocrine therapy and chemotherapy, the later of the two last systemic therapy dates was used to calculate the subsequent treatment cut-off date.

Breast cancer treatment identification rules

Medicare Benefits Schedule (MBS) and Australian Classification of Health Interventions (ACHI; 5) codes used for identifying breast cancer treatments include both current and historical codes. Due to limited granularity of Victorian Admitted Episodes Dataset (VAED) and Medicare data, human epidermal growth factor receptor 2 (HER2)-directed therapies (Anatomic Therapeutic Chemical [ATC] L01FD) such as trastuzumab were not separately captured but were recorded under the broader category of chemotherapy (ATC L01; 6).

Treatment	Identification rule
Breast-conserving surgery (excisions)	MBS codes: 31512, 31513, 31514, 31515, 31516
	VAED ACHI block-level items: 1744, 1745, 1746
Mastectomy	MBS codes: 31519, 31520, 31522, 31533, 31524, 31528, 31529
	VAED ACHI block-level items: 1747, 1748, 1749, 1750, 1751
Chemotherapy (7)	MBS codes:
	13915, 13918, 13921, 13924, 13927, 13930, 13933, 13936, 13945, 13950
	PBS ATC classification: Chapter L01 – antineoplastic agents
	VAED ACHI code-level items:
	9619600, 9619700, 9619800, 9619900, 9620000, 9620100, 9620200, 9620300, 9620400, 9620500, 9620600
	VAED ICD-10-AM code: Z51.1 – chemotherapy session for neoplasm
Endocrine therapy	PBS ATC classification: Chapter L02 – endocrine therapy
Radiation therapy	MBS codes:
	All codes under Category 3, Group T2 – Radiation oncology except Subgroup 7 – Radiation oncology treatment verification and Subgroup 8 – Brachytherapy planning and verification.
	VAED ACHI block-level items:
	1786, 1787, 1788, 1789, 1790, 1791, 1792, 1795
	VAED ICD-10-AM code: Z51.0 – radiotherapy session
	VRMDS:
	Encounters where primary site of cancer (i.e., site of origin) is breast and radiation therapy target site is breast or chest wall.

MBS = Medicare Benefits Schedule; VAED = Victorian Admitted Episodes Dataset; ACHI = Australian Classification of Health Interventions; PBS = Pharmaceutical Benefits Scheme; ATC = Anatomic Therapeutic Chemical; ICD-10-AM = International Classification of Diseases, tenth revision, Australian modification (8); VRMDS = Victorian Radiotherapy Minimum Dataset.

Figure 2. Directed acyclic graph of the relationship between OCP treatment alignment and survival.

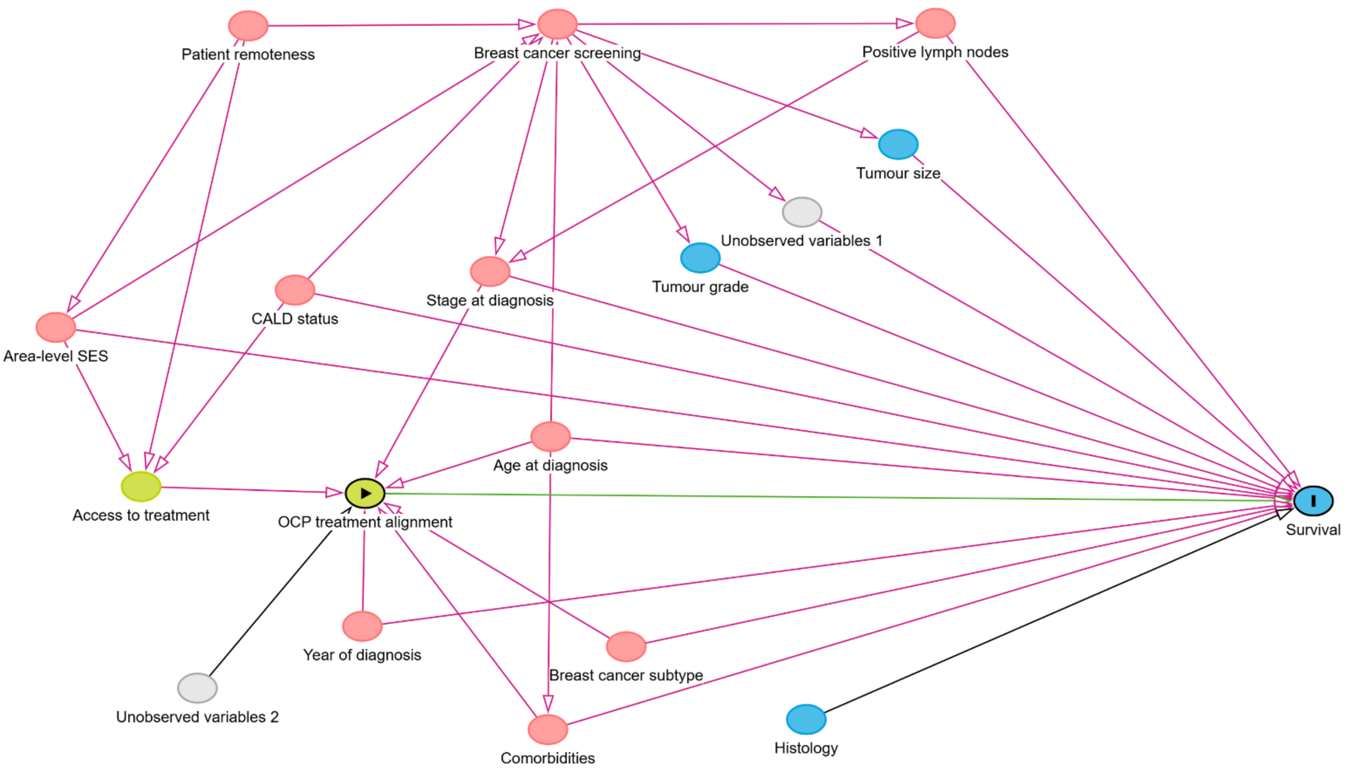

OCP = optimal care pathway; SES = socioeconomic status; CALD = culturally and linguistically diverse. Exposure of interest is optimal care pathway treatment alignment and outcome is survival. Red indicates confounding relationships. Blue indicates competing exposures. Grey indicates unobserved effects. Green indicates ancestral relationships to the main exposure of interest. Created using DAGitty (9).

Figure 3. Cumulative incidence curves of time-invariant study covariates.

A.

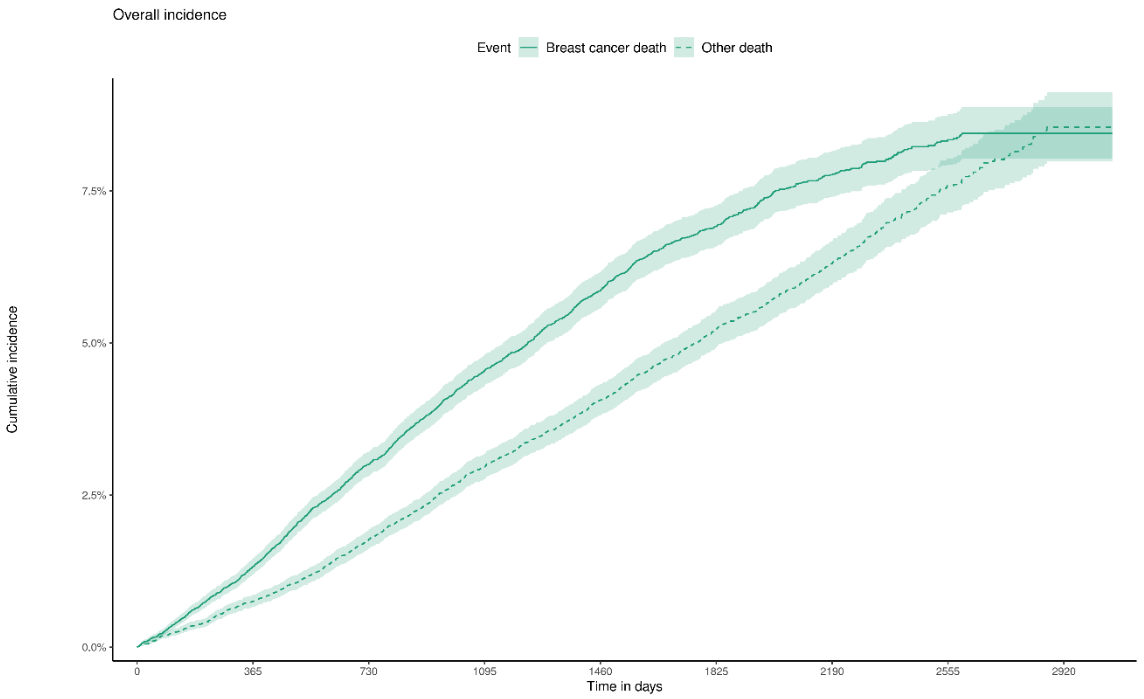

	0	365	730	1095	1460	1825	2190	2555	2920
Num. at risk	29591	28668	23808	19262	15023	11199	7592	4238	1152
Cum. events	0	615	1347	1971	2423	2752	2961	3089	3125
Cum. censors	0	309	4441	8361	12147	15669	19059	22274	25314

B.

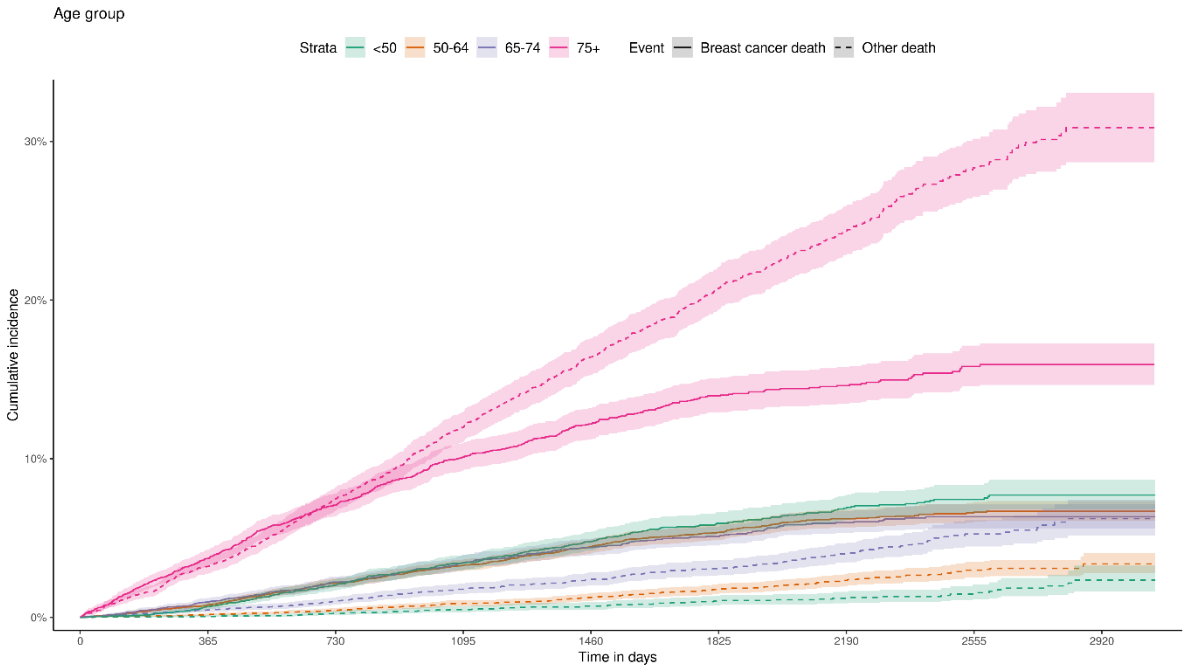

Number at risk (Cumulative events)

10816 (0)	10594 (105)	8947 (266)	7390 (397)	5901 (505)	4475 (584)	3089 (643)	1767 (671)	483 (676)
7330 (0)	7145 (105)	5915 (224)	4731 (335)	3679 (404)	2763 (454)	1806 (500)	942 (524)	242 (530)
5206 (0)	4794 (360)	3745 (723)	2818 (1024)	2038 (1235)	1412 (1388)	914 (1466)	480 (1528)	110 (1544)
6239 (0)	6135 (45)	5201 (134)	4323 (215)	3405 (279)	2549 (326)	1783 (352)	1049 (366)	317 (375)

C.

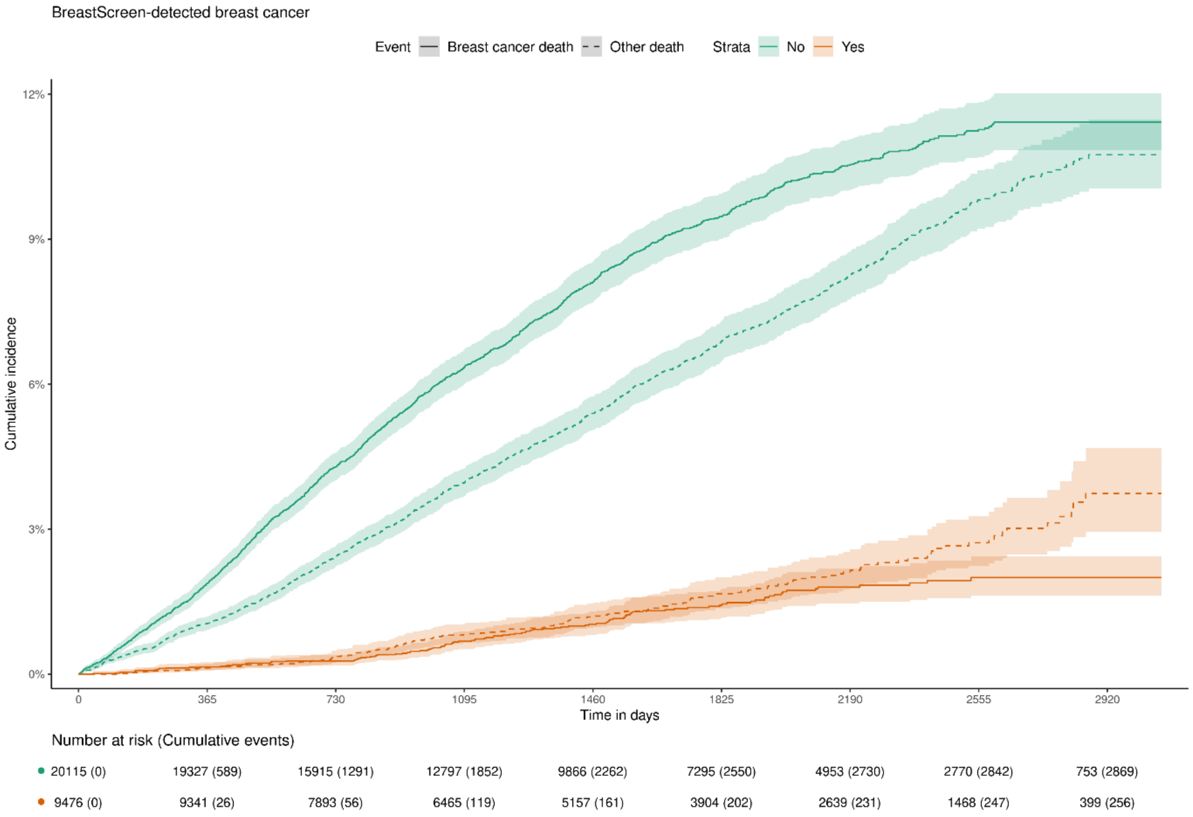
BreastScreen-detected breast cancer
Event
Breast cancer death
Other death
Strata
No
Yes
Cumulative incidence
12%
9%
6%
3%
0%
0
365
730
1095
1460
1825
2190
2555
2920
Time in days
Number at risk (Cumulative events)
20115 (0) 19327 (589) 15915 (1291) 12797 (1852) 9866 (2262) 7295 (2550) 4953 (2730) 2770 (2842) 753 (2869)
9476 (0) 9341 (26) 7893 (56) 6465 (119) 5157 (161) 3904 (202) 2639 (231) 1468 (247) 399 (256)

D.

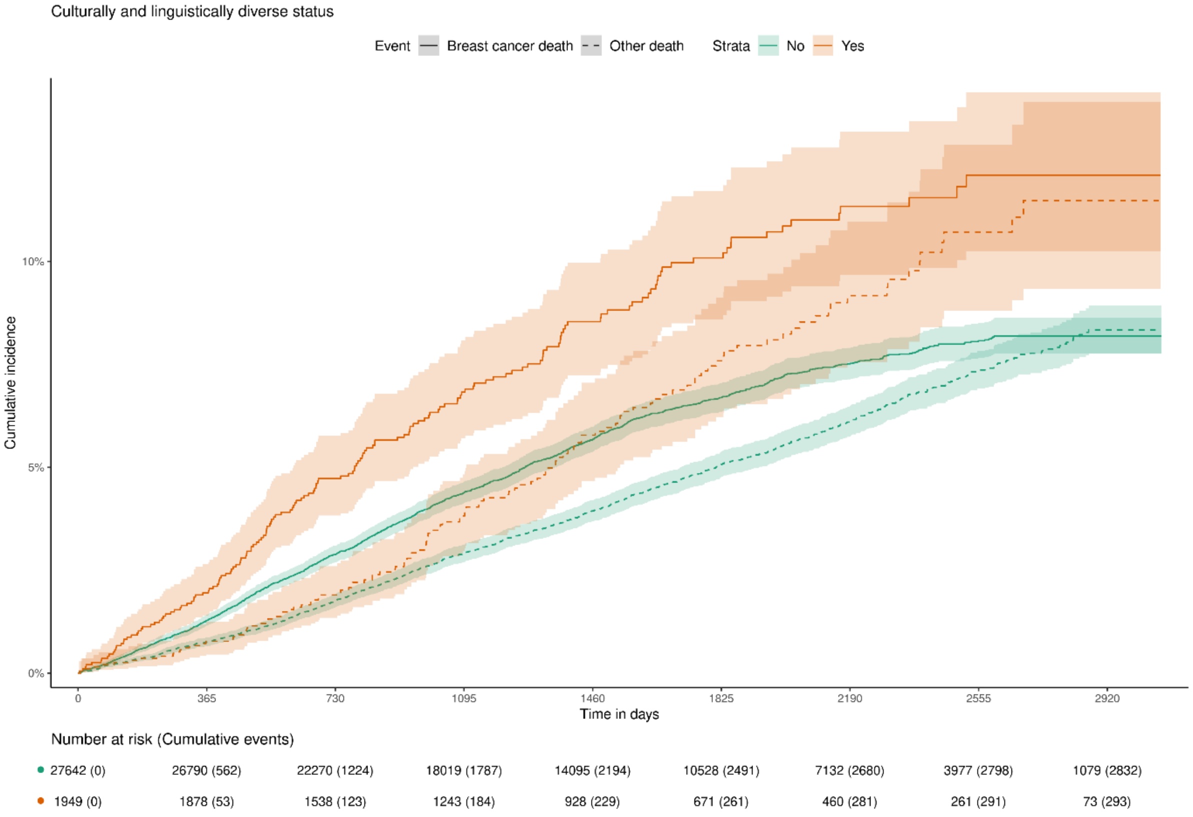
Culturally and linguistically diverse status
Event
Breast cancer death
Other death
Strata
No
Yes
Cumulative incidence
10%
5%
0%
0
365
730
1095
1460
1825
2190
2555
2920
Time in days
Number at risk (Cumulative events)
27642 (0) 26790 (562) 22270 (1224) 18019 (1787) 14095 (2194) 10528 (2491) 7132 (2680) 3977 (2798) 1079 (2832)
1949 (0) 1878 (53) 1538 (123) 1243 (184) 928 (229) 671 (261) 460 (281) 261 (291) 73 (293)

E.

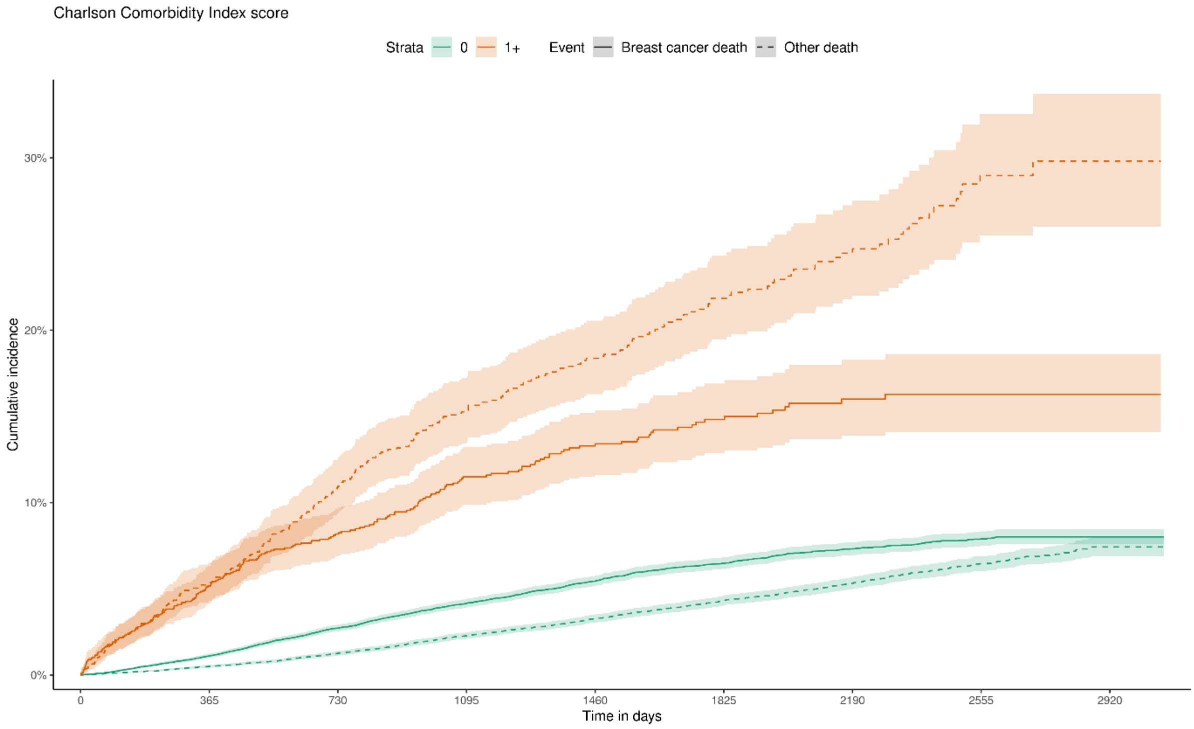

Number at risk (Cumulative events)

28022 (0)	27272 (450)	22747 (1058)	18476 (1591)	14441 (1995)	10822 (2289)	7358 (2478)	4128 (2593)	1123 (2628)
1569 (0)	1396 (165)	1061 (289)	786 (380)	582 (428)	377 (463)	234 (483)	110 (496)	29 (497)

F.

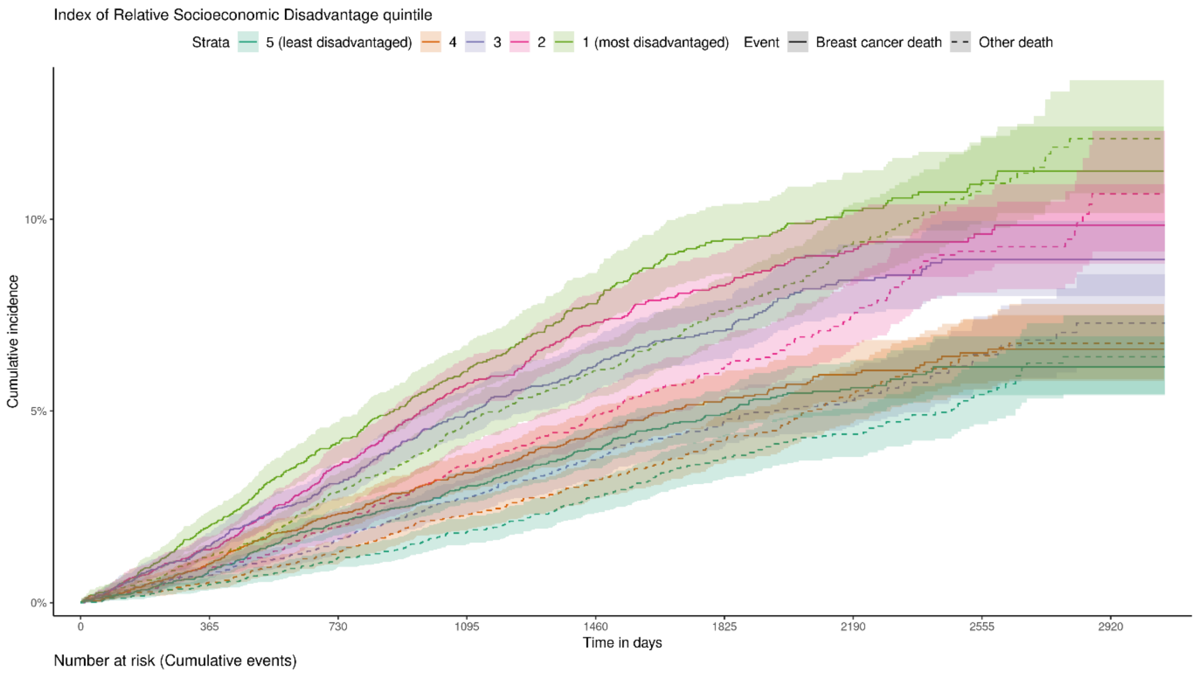

Number at risk (Cumulative events)

5589 (0)	5349 (179)	4398 (376)	3517 (533)	2711 (645)	1994 (737)	1355 (787)	721 (816)	198 (825)
5570 (0)	5392 (128)	4462 (294)	3589 (455)	2802 (559)	2076 (621)	1369 (667)	758 (696)	223 (705)
5740 (0)	5550 (126)	4645 (262)	3709 (391)	2858 (472)	2138 (526)	1453 (566)	803 (588)	210 (593)
5992 (0)	5839 (93)	4836 (207)	3978 (301)	3121 (377)	2341 (433)	1597 (472)	907 (495)	249 (499)
6700 (0)	6538 (89)	5467 (208)	4469 (291)	3531 (370)	2650 (435)	1818 (469)	1049 (494)	272 (503)

G.

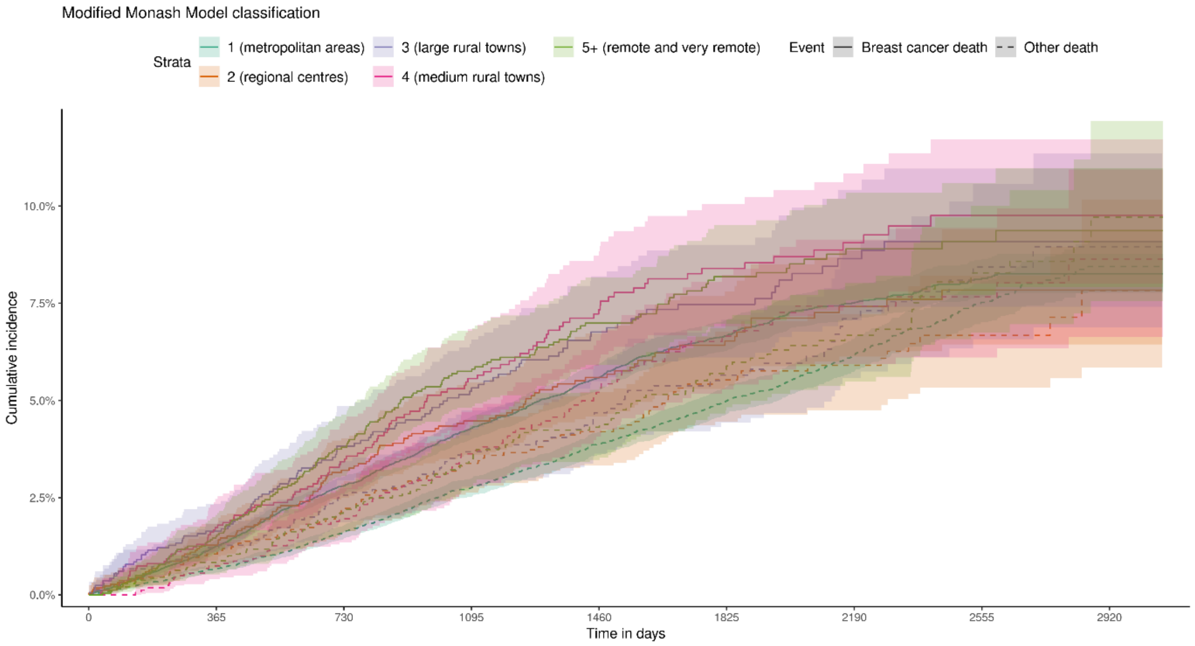

Number at risk (Cumulative events)

●	21735 (0)	21083 (421)	17564 (916)	14237 (1357)	11130 (1692)	8295 (1929)	5612 (2091)	3118 (2192)	834 (2219)
●	2191 (0)	2114 (51)	1708 (112)	1371 (152)	1073 (178)	786 (202)	534 (212)	306 (218)	84 (220)
●	1650 (0)	1586 (47)	1301 (100)	1066 (133)	813 (158)	614 (172)	413 (187)	233 (194)	60 (195)
●	1618 (0)	1565 (40)	1310 (83)	1031 (131)	797 (165)	606 (186)	431 (195)	247 (199)	79 (201)
●	2397 (0)	2320 (56)	1925 (136)	1557 (198)	1210 (230)	898 (263)	602 (276)	334 (286)	95 (290)

H.

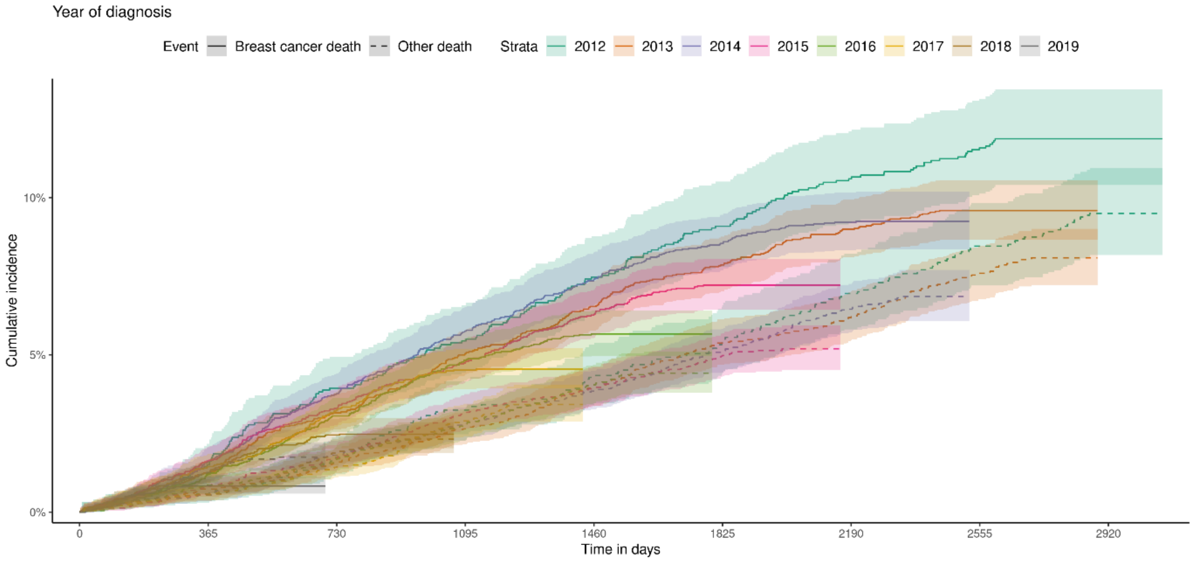

Number at risk (Cumulative events)

●	1727 (0)	1691 (36)	1629 (98)	1577 (150)	1524 (203)	1476 (251)	1424 (303)	1381 (346)	1152 (369)
●	3703 (0)	3630 (73)	3527 (176)	3419 (285)	3314 (389)	3213 (490)	3141 (563)	2857 (636)	0 (649)
●	3875 (0)	3791 (84)	3659 (216)	3545 (330)	3440 (436)	3346 (529)	3027 (607)	0 (619)	0 (619)
●	3880 (0)	3785 (95)	3687 (195)	3573 (308)	3484 (397)	3164 (473)	0 (479)	0 (479)	0 (479)
●	3956 (0)	3875 (82)	3770 (188)	3657 (300)	3261 (384)	0 (395)	0 (395)	0 (395)	0 (395)
●	4074 (0)	3992 (82)	3884 (191)	3491 (302)	0 (318)	0 (318)	0 (318)	0 (318)	0 (318)
●	4163 (0)	4088 (75)	3652 (180)	0 (193)	0 (193)	0 (193)	0 (193)	0 (193)	0 (193)
●	4213 (0)	3816 (88)	0 (103)	0 (103)	0 (103)	0 (103)	0 (103)	0 (103)	0 (103)

I.

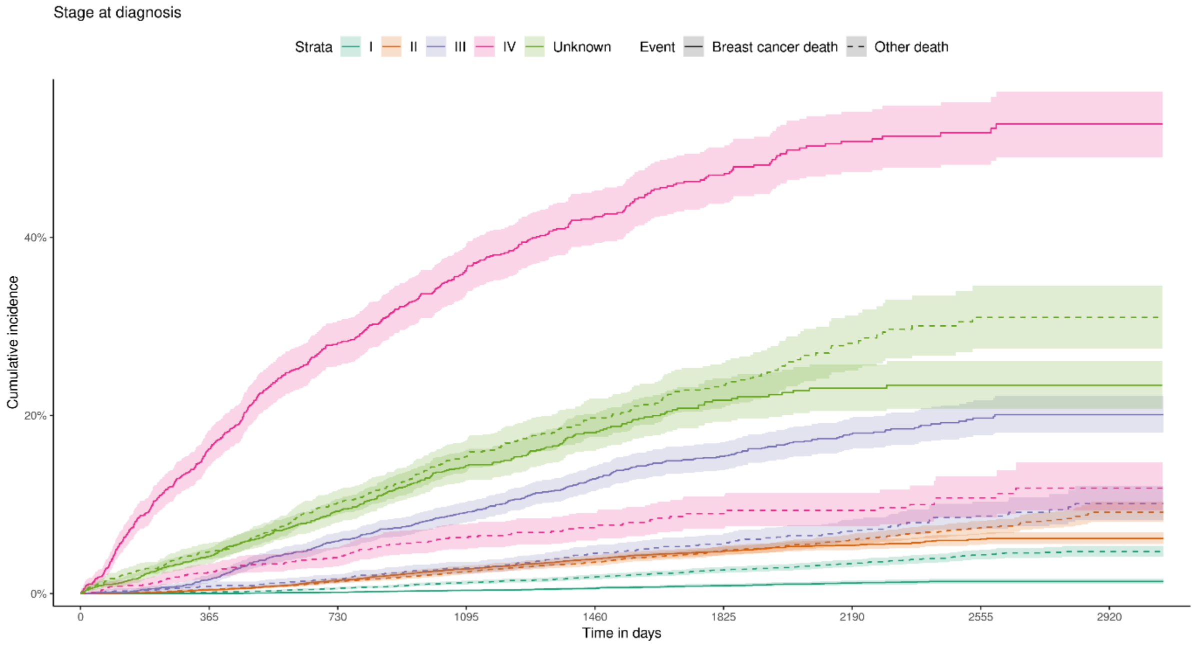

Number at risk (Cumulative events)

12679 (0)	12558 (24)	10753 (90)	9000 (174)	7319 (245)	5570 (317)	3862 (367)	2144 (403)	609 (409)
10768 (0)	10609 (94)	8910 (286)	7104 (486)	5489 (634)	4042 (748)	2705 (817)	1530 (867)	391 (886)
2717 (0)	2638 (65)	2205 (196)	1835 (296)	1416 (399)	1056 (457)	710 (501)	398 (527)	108 (534)
1299 (0)	1045 (243)	744 (405)	524 (510)	351 (567)	241 (606)	149 (626)	80 (633)	19 (637)
2128 (0)	1818 (189)	1196 (370)	799 (505)	448 (578)	290 (624)	166 (650)	86 (659)	25 (659)

J.

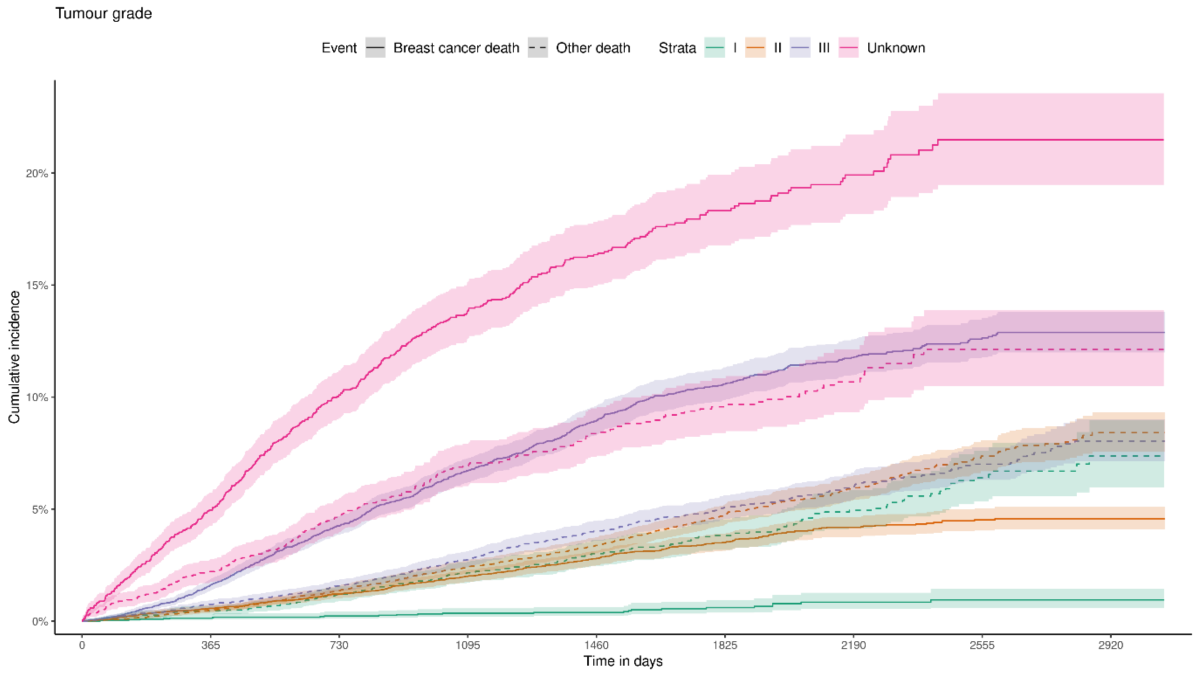

Number at risk (Cumulative events)

4437 (0)	4371 (26)	3717 (60)	3081 (97)	2471 (122)	1856 (145)	1225 (167)	669 (182)	184 (186)
12395 (0)	12150 (131)	10269 (303)	8397 (481)	6587 (619)	5030 (750)	3416 (838)	1937 (890)	558 (906)
9507 (0)	9204 (225)	7565 (528)	6090 (794)	4747 (1007)	3604 (1140)	2496 (1217)	1418 (1262)	349 (1278)
3252 (0)	2943 (233)	2257 (456)	1694 (599)	1218 (675)	709 (717)	455 (739)	214 (755)	61 (755)

K.

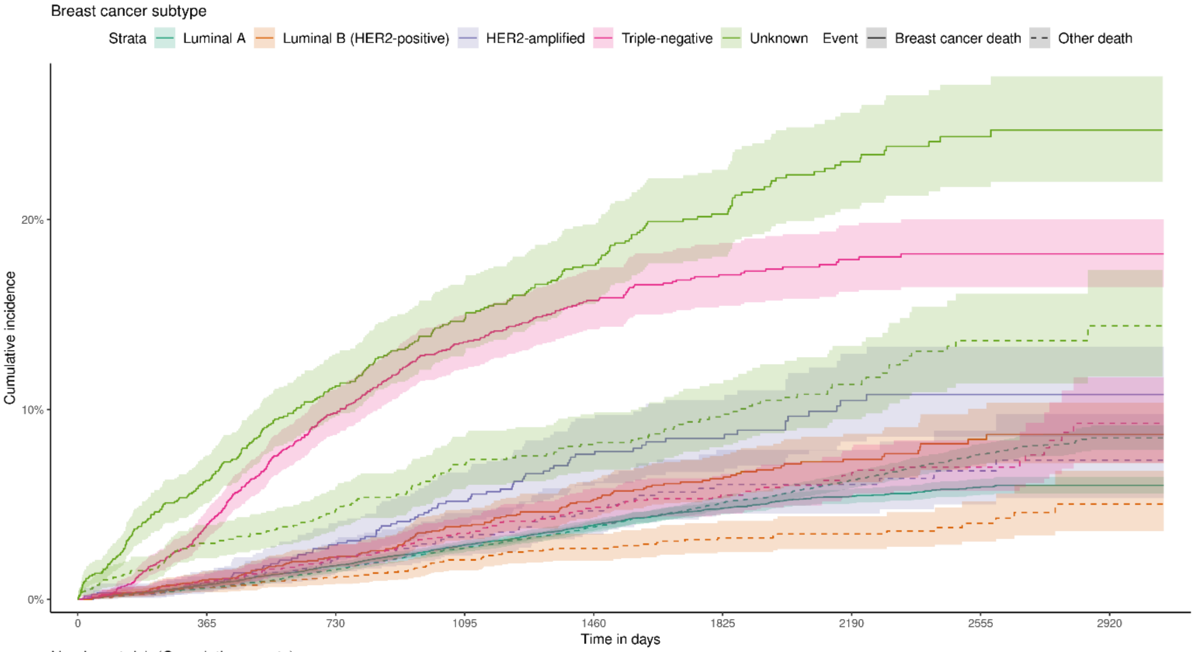

Number at risk (Cumulative events)

1161 (0)	1131 (19)	904 (54)	736 (84)	566 (112)	412 (125)	283 (133)	161 (136)	52 (137)
21933 (0)	21420 (299)	17879 (697)	14525 (1074)	11317 (1368)	8465 (1602)	5733 (1745)	3219 (1841)	877 (1864)
2549 (0)	2477 (42)	2064 (83)	1663 (132)	1305 (164)	998 (185)	678 (197)	362 (206)	84 (210)
2492 (0)	2344 (121)	1880 (287)	1471 (385)	1123 (442)	820 (467)	545 (484)	308 (488)	76 (494)
1456 (0)	1296 (134)	1081 (226)	867 (296)	712 (337)	504 (373)	353 (402)	188 (418)	63 (420)

L.

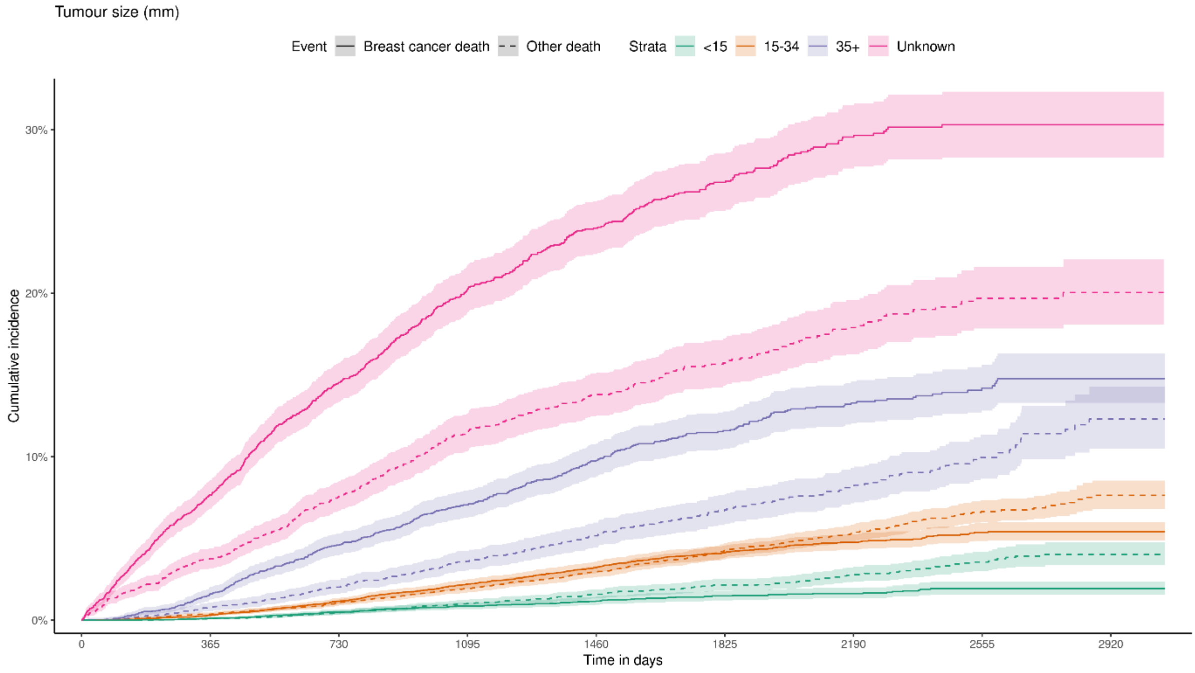

Number at risk (Cumulative events)

12214 (0)	12055 (79)	10300 (257)	8509 (442)	6768 (606)	5108 (747)	3479 (827)	1942 (886)	496 (899)
4170 (0)	4049 (100)	3361 (263)	2676 (396)	2010 (511)	1468 (583)	1013 (632)	573 (659)	151 (675)
9559 (0)	9476 (20)	8017 (83)	6538 (152)	5149 (204)	3860 (245)	2623 (270)	1469 (293)	431 (299)
3648 (0)	3088 (416)	2130 (744)	1539 (981)	1096 (1102)	763 (1177)	477 (1232)	254 (1251)	74 (1252)

M.

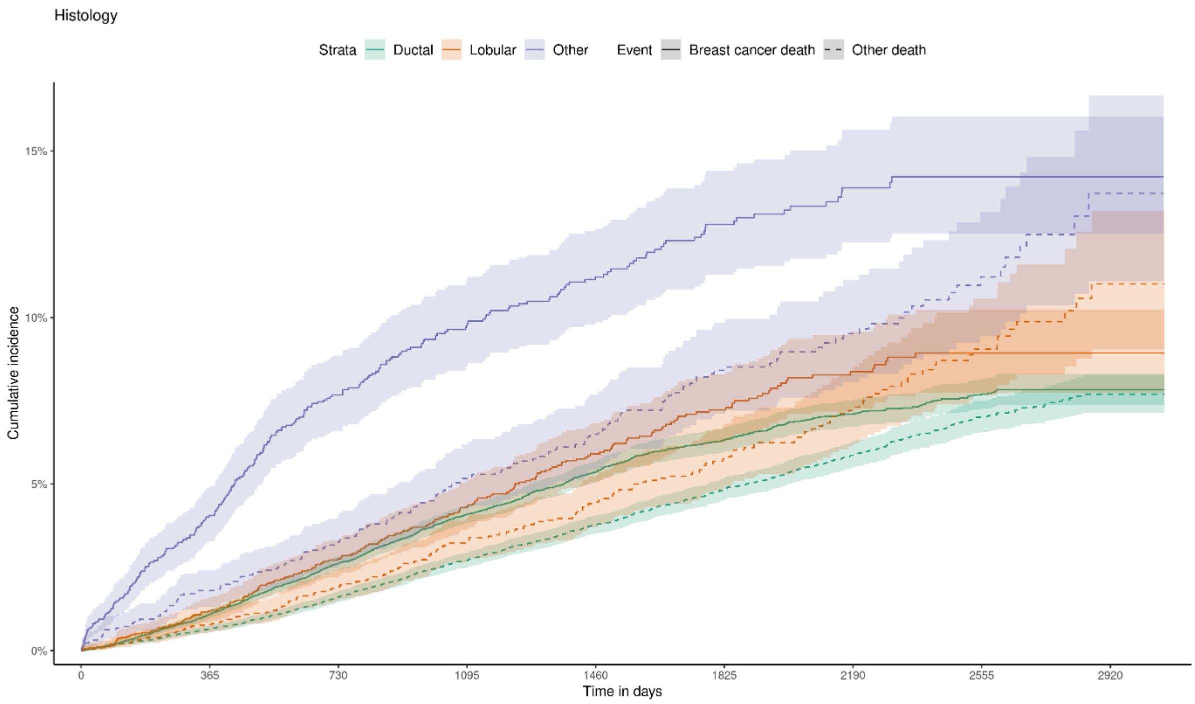

Number at risk (Cumulative events)

23833 (0)	23167 (415)	19237 (955)	15618 (1428)	12217 (1781)	9123 (2023)	6206 (2182)	3480 (2279)	933 (2302)
3539 (0)	3428 (70)	2867 (157)	2284 (238)	1747 (297)	1289 (344)	856 (376)	467 (396)	137 (403)
2219 (0)	2073 (130)	1704 (235)	1360 (305)	1059 (345)	787 (385)	530 (403)	291 (414)	82 (420)

N.

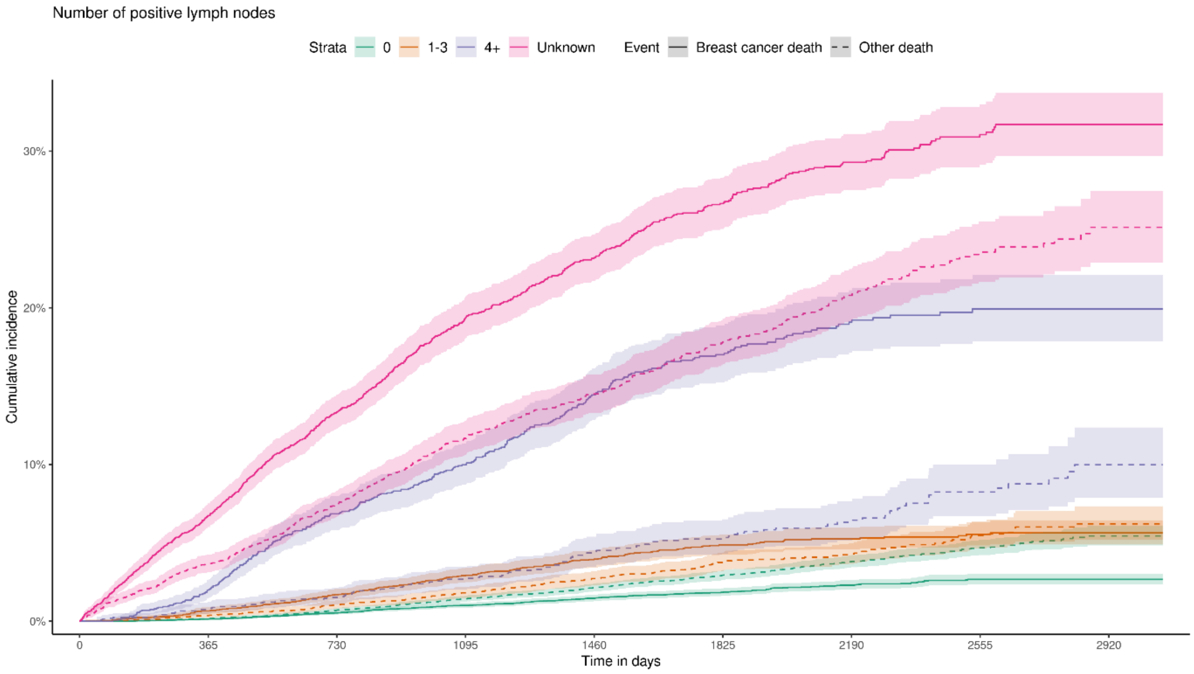

Number at risk (Cumulative events)

17240 (0)	17097 (51)	14530 (198)	11926 (358)	9411 (486)	7076 (588)	4813 (671)	2671 (720)	718 (734)
5900 (0)	5815 (59)	4973 (154)	4081 (247)	3233 (322)	2416 (381)	1660 (402)	963 (423)	266 (430)
2170 (0)	2102 (60)	1751 (175)	1449 (252)	1091 (344)	822 (388)	559 (416)	307 (433)	81 (438)
4281 (0)	3654 (445)	2554 (820)	1806 (1114)	1288 (1271)	885 (1395)	560 (1472)	297 (1513)	87 (1523)

HER2 = human epidermal growth factor receptor 2**.** Figures A to N show the cumulative incidence with 95% confidence intervals and risk tables for overall survival and each of the 13 time-invariant study variables presented in Table 1 of the main text (10). Risk tables show the cumulative number of events in each stratum as well as the number of patients at risk of experiencing the event after accounting for censoring and events that occurred up to that timepoint. Analyses were conducted on the original data.

Table 1. Stepwise regressions examining the association between breast cancer subtype and breast cancer-specific and overall survival.

Model specification	Breast cancer-specific death		All-cause (overall) death	
	aHR (95% CI)	LRT (*P*)	aHR (95% CI)	LRT (*P*)
Univariable				
Luminal A	1.00 (reference)		1.00 (reference)	
Luminal B (HER2-positive)	1.29 (1.08–1.53)		0.95 (0.83–1.10)	
HER2-amplified	1.75 (1.40–2.18)		1.40 (1.18–1.66)	
Triple negative	3.77 (3.35–4.23)		2.50 (2.27–2.75)	
Subtype + stage at diagnosis		< 0.001		< 0.001
Luminal A	1.00 (reference)		1.00 (reference)	
Luminal B (HER2-positive)	0.87 (0.72–1.04)		0.71 (0.61–0.81)	
HER2-amplified	1.11 (0.89–1.39)		0.99 (0.83–1.18)	
Triple negative	3.49 (3.10–3.94)		2.34 (2.12–2.58)	
Subtype + stage + tumour grade		< 0.001		< 0.001
Luminal A	1.00 (reference)		1.00 (reference)	
Luminal B (HER2-positive)	0.69 (0.57–0.82)		0.63 (0.55–0.73)	
HER2-amplified	0.83 (0.66–1.04)		0.86 (0.72–1.03)	
Triple negative	2.53 (2.22–2.88)		2.00 (1.80–2.23)	
Subtype + stage + grade + age group		< 0.001		< 0.001
Luminal A	1.00 (reference)		1.00 (reference)	
Luminal B (HER2-positive)	0.77 (0.64–0.92)		0.80 (0.69–0.92)	
HER2-amplified	0.93 (0.74–1.17)		1.07 (0.89–1.29)	
Triple negative	2.62 (2.30–2.98)		2.15 (1.93–2.40)	
Subtype + stage + grade + age + alignment		< 0.001		< 0.001
Luminal A	1.00 (reference)		1.00 (reference)	
Luminal B (HER2-positive)	0.74 (0.61–0.89)		0.75 (0.64–0.87)	
HER2-amplified	0.89 (0.70–1.14)		1.01 (0.83–1.24)	
Triple negative	2.46 (2.11–2.86)		1.96 (1.72–2.24)	

aHR = adjusted hazards ratios; CI = confidence interval; LRT = likelihood ratio test (11); HER2 = human epidermal growth factor receptor 2. The outcome was death. Luminal A was defined as either estrogen or progesterone receptor-positive (i.e., hormone receptor-positive) and human epidermal growth factor receptor 2-negative. Luminal B was defined as hormone receptor-positive and human epidermal growth factor receptor 2-positive. HER2-amplified was defined as hormone receptor-negative and human epidermal growth factor receptor 2-positive. Triple negative was defined as hormone receptor-negative and human epidermal growth factor receptor 2-negative. The univariable model was not adjusted. Analyses were conducted and pooled across 50 imputations. Likelihood ratio tests compared the present model against the previous model in a stepwise fashion.

Table 2. Sensitivity analyses of the effects of different criteria for optimal care pathway alignment on its associations with breast cancer-specific and overall survival.

A.

Model specification	Imputed data			Original data	
	1: study default (aHR; 95% CI)	2 (aHR; 95% CI)	3 (aHR; 95% CI)	4 (aHR; 95% CI)	5 (aHR; 95% CI)
All-time non-interaction model					
OCP alignment	0.77 (0.69, 0.86)	0.85 (0.76, 0.95)	0.85 (0.76, 0.95)	0.90 (0.80, 1.02)	0.80 (0.67, 0.94)
Time-limited non-interaction model					
1-year survival	0.71 (0.57, 0.90)	0.79 (0.62, 0.99)	0.79 (0.62, 1.00)	0.77 (0.58, 1.03)	0.36 (0.21, 0.62)
3-year survival	0.75 (0.65, 0.85)	0.84 (0.73, 0.96)	0.84 (0.73, 0.96)	0.88 (0.76, 1.03)	0.74 (0.60, 0.92)
5-year survival	0.75 (0.66, 0.84)	0.83 (0.73, 0.93)	0.83 (0.73, 0.93)	0.87 (0.77, 0.99)	0.75 (0.63, 0.89)
All-time interaction model					
Alignment: stage I	0.90 (0.60, 1.34)	0.90 (0.60, 1.34)	0.87 (0.59, 1.30)	1.04 (0.69, 1.57)	0.94 (0.57, 1.54)
Alignment: stage II	0.60 (0.50, 0.73)	0.83 (0.68, 1.01)	0.83 (0.68, 1.01)	0.82 (0.67, 1.00)	0.82 (0.64, 1.04)
Alignment: stage III	0.70 (0.58, 0.86)	0.71 (0.58, 0.86)	0.71 (0.58, 0.86)	0.82 (0.66, 1.02)	0.76 (0.58, 0.99)
Alignment: stage IV	0.97 (0.79, 1.19)	0.97 (0.79, 1.20)	0.97 (0.79, 1.20)	1.05 (0.83, 1.32)	0.74 (0.40, 1.35)

B.

Model specification	Imputed data			Original data	
	1: study default (aHR; 95% CI)	2 (aHR; 95% CI)	3 (aHR; 95% CI)	4 (aHR; 95% CI)	5 (aHR; 95% CI)
All-time non-interaction model					
OCP alignment	0.66 (0.60–0.71)	0.74 (0.68–0.81)	0.74 (0.68–0.81)	0.80 (0.73–0.88)	0.74 (0.65–0.83)
Time-limited non-interaction model					
1-year survival	0.61 (0.51–0.74)	0.70 (0.58–0.84)	0.70 (0.58–0.85)	0.70 (0.55–0.88)	0.46 (0.32–0.68)
3-year survival	0.62 (0.56–0.69)	0.72 (0.64–0.80)	0.72 (0.65–0.81)	0.78 (0.69–0.88)	0.70 (0.59–0.82)
5-year survival	0.64 (0.59–0.70)	0.72 (0.66–0.80)	0.73 (0.66–0.80)	0.79 (0.71–0.87)	0.71 (0.62–0.80)
All-time interaction model					
Alignment: stage I	0.61 (0.51–0.74)	0.61 (0.51–0.74)	0.62 (0.51–0.76)	0.77 (0.63–0.95)	0.76 (0.60–0.95)
Alignment: stage II	0.51 (0.45–0.58)	0.69 (0.60–0.80)	0.69 (0.60–0.80)	0.73 (0.63–0.84)	0.72 (0.61–0.85)
Alignment: stage III	0.67 (0.56–0.79)	0.66 (0.56–0.79)	0.66 (0.56–0.79)	0.79 (0.65–0.95)	0.75 (0.59–0.95)
Alignment: stage IV	0.97 (0.79–1.19)	0.97 (0.79–1.19)	0.97 (0.79–1.19)	1.01 (0.81–1.26)	0.71 (0.42–1.22)

aHR = adjusted hazards ratios; CI = confidence interval; OCP = optimal care pathway. Tables A and B respectively show, in adjusted hazards ratios, the associations between optimal care pathway alignment and breast cancer-specific and overall survival under different criteria for optimal care pathway alignment. Results for criteria 1 to 3 were from pooled analyses from imputed data. Results for criteria 4 were from analyses on the original data after excluding cases with missing cancer stage and subtype. Results for criteria 5 were from complete case analyses. 1) Default study criteria. Patients with stage III or IV cancer must adhere to a treatment pathway involving systemic therapy to be aligned. 2) Patients with stage II, III or IV cancer must adhere to a treatment pathway involving systemic therapy to be aligned. 3) Patients with stage II, III or IV cancer or patients with human epidermal growth factor receptor 2-positive or triple negative cancer must adhere to a treatment pathway involving systemic therapy to be aligned. 4) and 5) Identical criteria to criteria 1: patients with stage III or IV cancer must adhere to a treatment pathway involving systemic therapy to be aligned.

References

1. Cancer Council Victoria and Department of Health Victoria. Optimal care pathway for people with breast cancer. 2nd ed. Melbourne: Cancer Council Victoria, 2021. https://www.cancer.org.au/assets/pdf/breast-cancer-optimal-cancer-care-pathway (viewed Sep 2025).

2. Colleoni M, Litman HJ, Castiglione-Gertsch M, Sauerbrei W, Gelber RD, Bonetti M, Coates AS, Schumacher M, Bastert G, Rudenstam CM, Schmoor C, Lindtner J, Collins J, Thürlimann B, Holmberg SB, Crivellari D, Beyerle C, Neumann RL, Goldhirsch A . Duration of adjuvant chemotherapy for breast cancer: a joint analysis of two randomised trials investigating three versus six courses of CMF. *Br J Cancer* 2002; 86: 1705-1714.

3. Lerebours F, Cabel L, Pierga J-Y. Neoadjuvant endocrine therapy in breast cancer management: state of the art. *Cancers (Basel)* 2021; 13: 902.

4. Lai V, Hajjaj O, Le D, Shokoohi A, Chia S, Simmons C. Impact of wait time from neoadjuvant chemotherapy to surgery in breast cancer: does time to surgery affect patient outcomes? *Breast Cancer Res Treat* 2020; 184: 755-762.

5. Independent Health and Aged Care Pricing Authority. ICD-10-AM/ACHI/ACS eleventh edition. Sydney: IHACPA, 2022. https://www.ihacpa.gov.au/resources/icd-10-amachiacs-eleventh-edition (viewed Jun 2025).

6. World Health Organization Collaborating Centre for Drug Statistics Methodology. Guidelines for ATC classification and DDD assignment 2026. Oslo: WHO CCDSM, 2026. https://atcddd.fhi.no/filearchive/publications/2026_guidelines_for_atc_classification_and_ddd_assignment.pdf (viewed Feb 2026).

7. Tervonen HE, Creighton N, Zhao GW, Ng M, Currow DC. Capture of systemic anticancer therapy use by routinely collected health datasets. *Public Health Res Pract* 2020; 30: 3012004.

8. National Casemix and Classification Centre. The International Statistical Classification of Diseases and Related Health Problems, tenth revision, Australian modification. 8th ed. Sydney: National Casemix and Classification Centre, 2013.

9. Textor J, van der Zander B, Gilthorpe MS, Liskiewicz M, Ellison GT. Robust causal inference using directed acyclic graphs: the R package 'dagitty'. *Int J Epidemiol* 2016; 45: 1887-1894.

10. Kim HT. Cumulative incidence in competing risks data and competing risks regression analysis. *Clin Cancer Res* 2007; 13: 559-565.

11. Meng XL, Rubin DB. Performing likelihood ratio tests with multiply-imputed data sets. *Biometrika* 1992; 79: 103-111.

STROBE Statement—checklist of items that should be included in reports of observational studies

	Item No.	Recommendation	Page No.	Relevant text from manuscript
**Title and abstract**	1	(*a*) Indicate the study's design with a commonly used term in the title or the abstract	1	See *Design* subheading<br>"Retrospective population-based cohort study."
		(*b*) Provide in the abstract an informative and balanced summary of what was done and what was found	1	See *Design, Results* and *Conclusion* subheadings
**Introduction**				
Background/rationale	2	Explain the scientific background and rationale for the investigation being reported	3	Paragraph 4.<br>"In Australia, most OCP studies focus on feasibility and alignment. Only one Victorian population-based colon cancer study evaluated clinical outcomes associated with OCP alignment."
Objectives	3	State specific objectives, including any prespecified hypotheses	3	Paragraph 4.<br>"To address this gap in the evaluation of OCPs and outcomes, the present study investigated the relationship between alignment to the breast cancer OCP and survival."
**Methods**				
Study design	4	Present key elements of study design early in the paper	3-4	Paragraph 1.<br>"A retrospective population-based cohort study was conducted to quantify the association between alignment to the breast cancer OCP treatment step, and breast cancer-specific and overall survival in Victoria, Australia between 2012 and 2019 using linked administrative health data."
Setting	5	Describe the setting, locations, and relevant dates, including periods of recruitment, exposure, follow-up, and data collection	4	Paragraph 1.<br>"The study cohort identified adult Victorian women (female sex v. male) registered in the Victorian Cancer Registry (VCR) with invasive, unilateral breast cancer (C50, International Classification of Diseases, tenth revision, Australian modification) between 1 July 2012 and 31 December 2019."
Participants	6	(*a*) *Cohort study*—Give the eligibility criteria, and the sources and methods of selection of participants. Describe methods of follow-up<br>*Case-control study*—Give the eligibility criteria, and the sources and methods of case ascertainment and control selection.	4, 6, 16	See *Figure 1* for patient selection.<br>Paragraph 1 of *Statistical analysis* section.<br>"Patient follow-up began on VCR date of diagnosis (t0) and ended at death (event) or 30 November 2020 (censoring), whichever occurred first."

		Give the rationale for the choice of cases and controls<br>*Cross-sectional study*—Give the eligibility criteria, and the sources and methods of selection of participants		
		(*b*) *Cohort study*—For matched studies, give matching criteria and number of exposed and unexposed<br>*Case-control study*—For matched studies, give matching criteria and the number of controls per case	-	Not applicable. Patients were not matched across OCP alignment (exposure of interest).
Variables	7	Clearly define all outcomes, exposures, predictors, potential confounders, and effect modifiers. Give diagnostic criteria, if applicable	6, 17-20, 21-23	See *Table 1* and *Table 2* for table of covariates and *Supporting Information, figure 2* for directed acyclic graph. Paragraph 3 of *Statistical analysis* section.<br>"Multivariable time-varying Cox regressions were conducted to quantify the association between OCP alignment and survival in adjusted hazards ratio (aHRs) while controlling for time-invariant confounders and competing exposures such as cancer stage at diagnosis and age."
Data sources/ measurement	8*	For each variable of interest, give sources of data and details of methods of assessment (measurement). Describe comparability of assessment methods if there is more than one group	4, 17-20	See Paragraph 1 for description of data sources such as the Victorian Cancer Registry. "Available data included information on cancer diagnoses and clinical features, other patient medical conditions, patient area-level socioeconomic condition measured by the Index of Relative Socioeconomic Disadvantage, patient remoteness measured by the Modified Monash Model, mortality to 30 November 2020, breast screening and treatments."<br>See *Table 1* for description of measurement of each variable.
Bias	9	Describe any efforts to address potential sources of bias	6, 7	Paragraph 2 of *Statistical analysis* section.<br>"Protecting against immortal time bias, OCP alignment was a binary, time-varying covariate with all patients starting as not aligned at diagnosis unless they initiated treatment on the day of diagnosis."<br>See *Missing data and sensitivity analyses* section for handling of missing data and testing different criteria for OCP alignment.
Study size	10	Explain how the study size was arrived at	16	See *Figure 1* for study cohort selection.
Quantitative variables	11	Explain how quantitative variables were handled in the analyses. If applicable,	17-20, 21-23	Quantitative variables and their groupings can be found in *Table 1* (original data) and *Table 2* (multiple imputed data).

		describe which groupings were chosen and why		
Statistical methods	12	(*a*) Describe all statistical methods, including those used to control for confounding	6	*Statistical analysis* section.<br>"Multivariable time-varying Cox regressions were conducted to quantify the association between OCP alignment and survival in adjusted hazards ratio (aHRs) while controlling for time-invariant confounders and competing exposures such as cancer stage at diagnosis and age."<br>*Supporting Information, figure 2* shows a Directed Acyclic Graph which guided variable selection.
		(*b*) Describe any methods used to examine subgroups and interactions	6	Paragraph 4 of *Statistical analysis* section.<br>"As baseline risks of mortality and criteria for OCP alignment differed across cancer stages, post-hoc models with a stage alignment interaction term were fitted to assess for potential interactions."
		(*c*) Explain how missing data were addressed	7	See section *Missing data and sensitivity analyses.*<br>"Missing data were imputed with multiple imputation with chained equations. Fifty imputations of 30 iterations were performed. To aid performance of the imputation model, survival time, breast cancer-specific and all-cause death, and treatments received up to six-months post-diagnosis were included along with study variables."
		(*d*) *Cohort study*—If applicable, explain how loss to follow-up was addressed<br>*Case-control study*—If applicable, explain how matching of cases and controls was addressed<br>*Cross-sectional study*—If applicable, describe analytical methods taking account of sampling strategy	6	Paragraph 1 of *Statistical analysis* section.<br>"Patient follow-up began on VCR date of diagnosis (t0) and ended at death (event) or 30 November 2020 (censoring), whichever occurred first."
		(*e*) Describe any sensitivity analyses	7	See section *Missing data and sensitivity analyses* section.<br>"… sensitivity analyses were conducted to examine the effects of different criteria for OCP alignment on its associations with survival. A complete case analysis and an analysis excluding cases with missing cancer stage or subtype from the original dataset were also conducted."
**Results**				
Participants	13*	(a) Report numbers of individuals at each stage of study—eg numbers potentially	16	See *Figure 1* for study cohort selection.

		eligible, examined for eligibility, confirmed eligible, included in the study, completing follow-up, and analysed		
		(b) Give reasons for non-participation at each stage	16	See *Figure 1* for study cohort selection, including reasons for exclusion.
		(c) Consider use of a flow diagram	16	See *Figure 1* for study cohort selection flow diagram.
Descriptive data	14*	(a) Give characteristics of study participants (eg demographic, clinical, social) and information on exposures and potential confounders	7-8, 17-20, 21-23	See paragraph 1-4 of *Results* for description of study participant characteristics including patient demographic characteristics, follow-up time, OCP alignment rate and outcomes.<br>See *Table 1* and *Table 2* for outcomes associated with study participant characteristics.
		(b) Indicate number of participants with missing data for each variable of interest	7, 17-20	Paragraph 1.<br>"Missing clinical data ranged from 1,456 (4.9%) for cancer subtype to 2,128 (7.2%) for cancer stage and 4,281 (14.5%) for the number of positive lymph nodes. A complete case analysis would have excluded 6,704 (22.7%) patients."<br>See *Table 1* for number of participants with missing data for each variable in the original (i.e., non-imputed) data.
		(c) *Cohort study*—Summarise follow-up time (eg, average and total amount)	6, 7	*Statistical analysis* paragraph 1 of *Methods.*<br>"Patient follow-up began on VCR date of diagnosis (t0) and ended at death (event) or 30 November 2020 (censoring), whichever occurred first."<br>*Results* paragraph 2.<br>"Median follow-up was 1,481 days (IQR, 850-2,210 days)."
Outcome data	15*	*Cohort study*—Report numbers of outcome events or summary measures over time	7	Paragraph 2.<br>"Of 3,125 (10.6%) deaths, 1,745 (55.8%) had breast cancer registered as the underlying cause. Five-year breast cancer-specific and overall survival was 93.1% (95% confidence interval [CI], 92.7%-93.4%) and 87.9% (95% CI, 87.4%-88.3%) respectively."
		Case-control study—Report numbers in each exposure category, or summary measures of exposure	-	Not applicable.
		Cross-sectional study—Report numbers of outcome events or summary measures	-	Not applicable.
Main results	16	(*a*) Give unadjusted estimates and, if applicable, confounder-adjusted estimates and their precision (eg, 95%	24-25	See *Figure 2* for univariable (i.e., unadjusted) and confounder-adjusted association of OCP alignment and survival.

		confidence interval). Make clear which confounders were adjusted for and why they were included		"The outcome was death. All models were fully adjusted with time-invariant study variables from Table 2 except the univariable model. Confidence intervals for the joint marginal effects of optimal care pathway alignment across cancer stages were manually calculated from the covariance matrix."
		(*b*) Report category boundaries when continuous variables were categorized	21-23	See *Table 2* for age group, tumour size and number of positive lymph node categories.
		(*c*) If relevant, consider translating estimates of relative risk into absolute risk for a meaningful time period	7, 21-23, 24-25	Paragraph 2.<br>"Five-year breast cancer-specific and overall survival was 93.1% (95% confidence interval [CI], 92.7%-93.4%) and 87.9% (95% CI, 87.4%-88.3%) respectively."<br>See *Table 1* for five-year survival by study covariates.<br>Relative risk (hazards ratios) is presented in *Figure 2.*
Other analyses	17	Report other analyses done—eg analyses of subgroups and interactions, and sensitivity analyses	8, 24-25	See paragraphs 2 and 3 of *Associations with survival* section, *Figure 2,* and *Supporting Information, table 2* for discussion of interaction effects and sensitivity analyses.
**Discussion**				
Key results	18	Summarise key results with reference to study objectives	8-9	Paragraph 1.<br>"Examining the records of nearly 30,000 Victorian women with breast cancer diagnosed between 2012 and 2019, we found that alignment to the high-level OCP treatment type and timing was associated with significantly lower risks of breast cancer-specific death and all-cause death compared with non-alignment. These associations were relatively stable over time since diagnosis but varied by cancer stage at diagnosis."
Limitations	19	Discuss limitations of the study, taking into account sources of potential bias or imprecision. Discuss both direction and magnitude of any potential bias	9-10, 11	See paragraphs 3 and 4 for discussion of potential residual confounding and sources of confounding.<br>"Nevertheless, a potentially greater association between OCP alignment and overall survival than breast cancer-specific survival may indicate residual confounding, or an association with healthcare pathways beyond cancer."<br>See paragraphs 1 and 2 of *Limitations* section for discussion of potential imprecision associated with relatively short follow-up time, missing data imputation and non-censoring of emigrated study participants.<br>"Our study could not censor those who emigrated from Victoria. The under-ascertainment of in-hospital treatments

				across borders is likely not trivial. However, since survival is high in our cohort, under-assignment of OCP-aligned time for emigrated and border patients is likely to lead to under-estimations of the associations between OCP alignment and survival.”
Interpretation	20	Give a cautious overall interpretation of results considering objectives, limitations, multiplicity of analyses, results from similar studies, and other relevant evidence	9, 11	Paragraph 1.<br>“Overall, our population-level findings in Victoria point to a survival benefit associated with alignment to the OCP after accounting for measured potential confounding factors.”<br>See also *Conclusion.*
Generalisability	21	Discuss the generalisability (external validity) of the study results	9	Paragraph 1.<br>“Overall, our population-level findings in Victoria point to a survival benefit associated with alignment to the OCP after accounting for measured potential confounding factors.”
**Other information**				
Funding	22	Give the source of funding and the role of the funders for the present study and, if applicable, for the original study on which the present article is based	-	Funding information will be entered at time of submission. The present article is an original study.

*Give information separately for cases and controls in case-control studies and, if applicable, for exposed and unexposed groups in cohort and cross-sectional studies.

Note: An Explanation and Elaboration article discusses each checklist item and gives methodological background and published examples of transparent reporting. The STROBE checklist is best used in conjunction with this article (freely available on the Web sites of PLoS Medicine at http://www.plosmedicine.org/, Annals of Internal Medicine at http://www.annals.org/, and Epidemiology at http://www.epidem.com/). Information on the STROBE Initiative is available at www.strobe-statement.org.
